# Wafer-scale integration of stretchable semiconducting polymer microstructures via capillary gradient

**DOI:** 10.1038/s41467-021-27370-w

**Published:** 2021-12-02

**Authors:** Yuchen Qiu, Bo Zhang, Junchuan Yang, Hanfei Gao, Shuang Li, Le Wang, Penghua Wu, Yewang Su, Yan Zhao, Jiangang Feng, Lei Jiang, Yuchen Wu

**Affiliations:** 1 Key Laboratory of Bio-inspired Materials and Interfacial Science, Technical Institute of Physics and Chemistry, Chinese Academy of Sciences, Beijing, 100190 P. R. China; 2 College of Chemistry, Jilin University, Changchun, 130012 P. R. China; 3 Beijing Key Laboratory of Lightweight Multi-Functional Composite Materials and Structures, Institute of Advanced Structure Technology, Beijing Institute of Technology, Beijing, 100081 P. R. China; 4 State Key Laboratory of Nonlinear Mechanics, Institute of Mechanics, Chinese Academy of Sciences, 100190 Beijing, P. R. China; 5 Department of Biomedical Engineering, Southern University of Science and Technology, Shenzhen, Guangdong 518055 P. R. China; 6 Department of Materials Science, Fudan University, Shanghai, 200433 P. R. China; 7 Department of Chemical and Biomolecular Engineering, National University of Singapore, Singapore, 117585 Singapore

**Keywords:** Electronic devices, Electronic devices

## Abstract

Organic semiconducting polymers have opened a new paradigm for soft electronics due to their intrinsic flexibility and solution processibility. However, the contradiction between the mechanical stretchability and electronic performances restricts the implementation of high-mobility polymers with rigid molecular backbone in deformable devices. Here, we report the realization of high mobility and stretchability on curvilinear polymer microstructures fabricated by capillary-gradient assembly method. Curvilinear polymer microstructure arrays are fabricated with highly ordered molecular packing, controllable pattern, and wafer-scale homogeneity, leading to hole mobilities of 4.3 and 2.6 cm^2^ V^−1^ s^−1^ under zero and 100% strain, respectively. Fully stretchable field-effect transistors and logic circuits can be integrated in solution process. Long-range homogeneity is demonstrated with the narrow distribution of height, width, mobility, on-off ratio and threshold voltage across a four-inch wafer. This solution-assembly method provides a platform for wafer-scale and reproducible integration of high-performance soft electronic devices and circuits based on organic semiconductors.

## Abstract (web summary)

Though stretchable semiconducting polymers are advantageous for electronic applications requiring low cost, the low performance of patterned microstructures remains a challenge. Here, the authors realize high mobility, stretchable polymer microstructures via a capillary-gradient assembly method.

## Introduction

Stretchable semiconducting materials are of great interest for soft and deformable electronics toward applications such as physiological monitoring^1^, bioelectronic interfaces^2^, robotics^3^, and implantable biomedical devices^4,5^. Mechanical flexibility is feasible by structural engineering of rigid inorganic semiconductors^6,7^ and employing intrinsically elastic polymers^8^. For instance, ultrathin nanomembranes^9^, nanowires^10^, 3D mesostructures^11^, and kirigami patterns^12^ have rendered rigid inorganics, such as silicon^6^ and III–V semiconductors^13,14^, with mechanical flexibility, but the stretchability is restricted by their intrinsically low fracture strain (~1% for silicon)^15^. Semiconducting polymers with inherently low modulus and solution processability afford a competitive approach to realize scalable fabrication of flexible devices with enhanced stretchability and high integration density. However, trade-offs between electronic performances and mechanical flexibility restrict the practical applications of semiconducting polymers. The fundamental issue is that considerable stretchability requires deformable components, such as flexible side chains/backbones^16^ and elastomer blends^17^, while high mobility demands a rigid π-conjugated molecular structure and ordered packing to suppress the conformational and energetic disorder^18–20^. Therefore, intrinsically stretchable polymers usually possess relatively low carrier mobility originating from a defect- and disorder-induced carrier localization and high-mobility rigid polymers experience serious degradation of electronic performances under high deformation^21^.

The concept of geometry engineering, which has been extensively studied to create stretchable wavy^22,23^, fractal^24,25^, and kirigami geometries^21,26^ based on inorganic materials, is hopeful to accommodate the challenge in the fabrication of high-mobility and stretchable semiconducting polymers. In striking contrast to inorganics with versatile top-down lithography techniques, solution-processing methods, which are regarded with unique advantages of low cost and high productivity^27,28^, involve an explicit compromise between stretchability and electronic performances for the patterning of stretchable polymer microstructures. Specifically, precise fabrication of complex microstructures demands the pattern of liquid films or droplets in high resolution, which poses a challenge to attain ordered molecular packing due to the uncontrollable microfluidic dynamics and mass transport^29–31^. High crystallinity and ordered packing of polymer molecules are feasible by introducing directional microfluids in the assembly process^32,33^, while this strategy presents restricted versatility for microstructure fabrication. In addition, wafer-scale integration of desired microstructures with ordered molecular packing has not yet been realized in the solution process. Reaching high packing order, stretchability, scalability, and solution processibility simultaneously will open up access to high-performance and low-cost soft electronic devices.

In this work, we develop a platform to fabricate stretchable polymer microstructures by steering capillary gradient for realizing ordered microfluid dynamics and precise patterning simultaneously. High crystallinity, complicated curvilinear geometries, and wafer-scale homogeneity are simultaneously achieved in polymer microstructures by optimizing the capillary-gradient-induced three sequential stages: breaking and trapping of liquids into micro-reservoirs, directional liquid transport into capillary bridges, and confined growth of polymer structures in capillary bridges. Due to the curvilinear structures with ordered molecular packing, high stretchability and carrier mobility are realized simultaneously based on these curvilinear structures. High mobilities of 4.3 and 2.1 cm^2^ V^−1^ s^−1^ are realized by using high-*k* Al_2_O_3_ and stretchable organic dielectrics, respectively. Based on a fully stretchable device configuration with solution-processed semiconducting channels, gate dielectrics, and electrodes, 92% preservation of mobility after 1000 stretch-release cycles under 50% strain and direct integration of 2000 stretchable transistors with a device density of 400 cm^−2^ are achieved. Wafer-scale uniformity is further demonstrated by a narrow distribution of height, width, mobility, on-off-ratio, and threshold voltage by careful statistics over 4000 sites on a 4-in. wafer.

## Results

### Capillary-gradient-mediated assembly of curvilinear polymer architectures

**Fig. 1:**
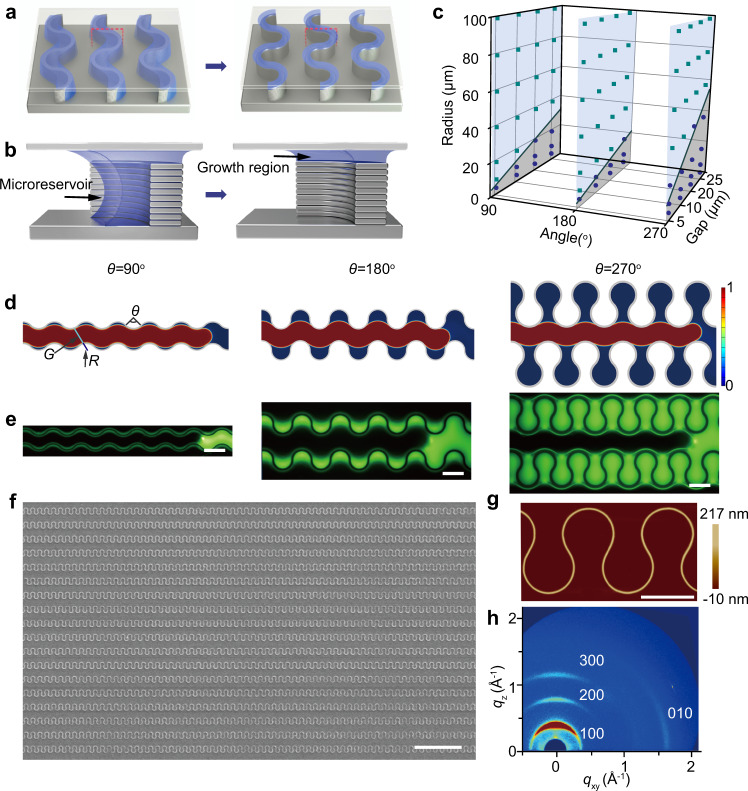
Capillary-gradient manipulation for the fabrication of ordered curvilinear polymer microstructures. **a** Schematic illustrations of dewetting and assembling processes of curvilinear polymer microstructures using a capillary-gradient assembly method. **b** Corresponding cross-sectional view schemes of (**a**). **c** Liquid trapping in microreservoirs as functions of arc radius *R*, arc angle *θ*, and minimum gap distance *G* of micropillars. The symbols of blue dots and green squares represent successful and unsuccessful trapping of liquid, respectively. The solid lines highlight the calculated threshold gap distances (see details in Supplementary Note 3). **d**, **e** Simulations and fluorescence microscope images of the liquid trapping in inner arc edge of the micropillar with central arc angle *θ* of 90^o^, 180^o^, and 270^o^. **f** Representative SEM image of large-area curvilinear polymer microstructure arrays with *θ* = 270^o^. **g** AFM topography and height diagram of a curvilinear P3HT microstructure with *θ* = 270^o^. **h** GIWAXS pattern of curvilinear P3HT microstructure arrays with the central arc angle *θ* = 270^o^, indicating the edge-on stacking of polymer chains. Scale bars: **e** 10 μm, **f** 100 μm, **g** 10 μm.

The capillary gradient is realized by employing programmable topographical templates with curvilinear micropillars fabricated by lithography and reactive-ion etching (Supplementary Fig. 1). The geometrical parameters of curvilinear micropillars include central angle *θ*, curvature radius *R*, and inter-pillar distance *G* (Supplementary Fig. 2), which can be optimized to achieve scalable fabrication of high-quality curvilinear structures. Central angle *θ* was designed as 90^o^, 180^o^, and 270^o^ to accommodate the fabrication of curvilinear microstructures with different stretchability. Assembly of polymer structures was performed by confining a liquid thin film of precursor solution between a topographical template and a target substrate (see Supplementary Fig. 3 and Method). Through in situ fluorescence microscopy observations, a sequential dewetting process can be observed in confined liquid films: receding of liquid firstly occurs between the curvilinear micropillars, resulting in localization of liquid into an inner arc to form discrete microreservoirs; with further evaporation of liquid, directional dewetting from microreservoirs to tops of micropillars yields pinned capillary bridges; finally, the confined growth of polymers in capillary bridges produces curvilinear microstructures (see Schematic illustration in Fig. 1a, b, Supplementary Fig. 3 and time-sequential optical micrographs in Supplementary Figs. 4–6). This process is driven by the gradient of capillary pressure, which can be expressed as^34^

1 $$P=-\frac{2\gamma\, {{{{{\rm{cos }}}}}}\,{\theta }_{Y}}{R}$$

where *γ* is the surface tension of the liquid, *θ*_Y_ is the contact angle, *R* is the equivalent radius of capillary. Driven by capillary pressure, liquid tends to concentrate in the position with a smaller capillary size (i.e., *R*) in the evaporation process. By rationally designing the geometrical parameters of curvilinear micropillars, directional transport of liquid can occur from the gaps between micropillars to microreservoirs, which finally localize on the tops of micropillars to form capillary bridges. For the dewetting process from microreservoirs to the tops of micropillars, we observed near 100% formation of capillary bridges, which can be attributed to the large capillary–pressure contrast between the microreservoirs and capillary bridges. The height of microreservoirs is *ca*. 20 μm, while capillary bridges on the tops of micropillars feature a sub-micrometer height, yielding over 20-fold higher capillary pressure in capillary bridges. The lattice Boltzmann method simulations also validate the directional liquid transport from microreservoirs to capillary bridges (Supplementary Fig. 7).

Successful liquid dewetting from inter-micropillar gaps to microreservoirs is crucial for the uniformity of as-prepared polymer microstructures at a long-range. Efficient trapping of liquid in microreservoirs enables nearly equivalent liquid volume and mass of polymer molecules at each arc (Fig. 1d, e), resulting in polymer microstructures with homogeneous size (Fig. 1f). In contrast, broad size distribution of polymer microstructures can be observed in the system without trapping of liquid in microreservoirs (Supplementary Fig. 8), which is caused by the fluctuation of polymer concentration in the dewetting process. The concentration of polymers firstly increases with the evaporation of solvents and then sustains a supersaturated state with the deposition of polymer microstructures, which finally decreases with the exhaustion of polymers in solution.

Given that efficient trapping of liquid into microreservoirs is significant for the long-range uniformity of polymer microstructures, we carefully studied the liquid dewetting in curvilinear micropillars with different geometrical parameters by experimental observations and computational fluidic dynamics (CFD) simulations. The dewetting dynamics were observed in micropillars with central angle *θ* of 90^o^, 180^o^, and 270^o^, the curvature radius of arc from 5 to 100 μm, and interpillar distance from 5 to 20 μm (Supplementary Figs. 9 and 10). According to the criteria of trapped liquid in microreservoirs, the dewetting results are summarized in Fig. 1c. These results indicate that increased interpillar gap distance *G* and decreased curvature radius *R* benefit the trapping of liquid into microreservoirs (Fig. 1c), which can be understood as high local capillary pressure in microreservoir region steers the liquid trapping. To develop a better understanding of this trapping process, CFD simulations were carried out (Supplementary Fig. 11, Supplementary Movie 1). According to our analysis, liquid trapping occurs when the length of the liquid front, which is proportional to interpillar gap distance *G*, is larger than the opening distance of curvilinear arc, which is a function of curvature radius *R* and central angle *θ* (see details in Supplementary Note 1). A threshold gap distance for liquid trapping can be calculated, shown as the solid lines in Fig. 1c. The calculated threshold gap distance is proportional to the curvature radius *R*, which is well consistent with the experimental results. The impacts of the rheological property of polymer solutions on the dewetting and polymer assembly are also evaluated by harnessing solvents with different viscosities (Supplementary Fig. 12). All polymer solutions present the transition from the continuous liquid film via trapped liquids in micro-reservoirs to pinned capillary bridges in the dewetting process, yielding curvilinear polymer microwire arrays.

By rational design of the micropillars, sequential dewetting from interpillar gaps via microreservoirs to capillary bridges boosts the large-scale fabrication of curvilinear polymer microstructures with long-range uniformity. A presentative semiconducting polymer, poly(3-hexylthiophene) (P3HT) were firstly employed to demonstrate the fabrication of curvilinear microstructures. As shown in scanning electron microscopy (SEM) images (Fig. 1f, Supplementary Fig. 13), the as-prepared P3HT curvilinear microstructures with different geometrical parameters exhibit homogeneous size and smooth surface. The capillary-gradient method is effective for the construction of structures on the sub-micrometer scale because more efficient liquid trapping occurs in nanopillars with smaller capillaries. To demonstrate the validity of capillary gradient for the assembly of sub-micrometer structures, we fabricated curvilinear polymer nanowires with *ca*. 150 nm in width and 2 μm in arc diameter by using a corresponding nanopillar template (Supplementary Fig. 14), yielding an integrated density of 6.25 million periods of arcs per square centimeter. The long-range uniformity and reproducibility of curvilinear structures are demonstrated by optical micrographs with different magnification (Supplementary Figs. 15–17). AFM topography illustrates that a curvilinear microstructure exhibits a uniform height of *ca*. 270 nm, the width of *ca*. 600 nm and a smooth top surface with a low root mean square roughness of 0.436 nm (Fig. 1g). The crystallinity and crystallographic orientation of P3HT microstructures were determined by grazing-incidence wide-angle X-ray scattering (GIWAXS) (Fig. 1h). GIWAXS pattern of P3HT curvilinear microstructures shows a series of rings along the *q*_z_ axis, which can be assigned to the (*h*00) reflections of the lamella layer structure. The diffraction ring lying on the *q*_xy_ axis, corresponding to a facet with a distance of 3.7 Å, can be attributed to the (010) reflections of *π*–*π* stacking^35^. This result indicates that the P3HT molecules exhibit a pure edge-on packing configuration with *π–π* stacking direction along the conductive channel, which benefits the charge-carrier transport in organic field-effect transistors (OFETs). The high crystallinity of polymers is underpinned by the regulated capillary microfluids and directional mass transport in capillary bridges, thus leading to highly ordered packing of molecules^36,37^. In contrast, spin-coated thin films show small grains with disordered molecular packing and low crystallinity, which are reflected by the SEM image and GIWAXS pattern (Supplementary Fig. 18).

The curvilinear microstructures are also demonstrated on two additional conjugated polymers, p-type poly[2,5-bis(2-decyltetradecyl)pyrrolo[3,4-c]pyrrole-1,4(2H,5H)-dione-alt-5,5′-di(thiophen-2-yl)-2,2′-(E)-2-(2-(thiophen-2-yl)vinyl)thiophene] (PDVT-10) and n-type poly{[N,N′-bis(2-octyldodecyl)-naphthalene-1,4,5,8-bis(dicarboximide)-2,6-diyl]-alt-5,5′-(2,2′-bithiophene)} (P(NDI2OD-T2)). High-quality curvilinear microstructures with smooth surfaces and homogeneous sizes are demonstrated. High crystallinity is demonstrated by sharp diffraction rings in GIWAXS patterns (Supplementary Fig. 19). To precise tune the size of the curvilinear structures, the influence of arc angle, arc radius, and precursor concentration on the size of microstructures were investigated. The curvilinear microstructures with arc angle *θ* = 270^o^ and radius from 5 to 100 μm are presented in Supplementary Fig. 20a. The size of the curvilinear microstructures is proportional to the volume of semiconducting polymer solution trapped in each microreservoir, which can be demonstrated by large width on the position with large microreservoir volume (Supplementary Fig. 20b). The width and height are proportional to the concentration of the precursor solution and radius of arc angle, which is demonstrated by theoretical analysis and experimental results (Supplementary Note 2, Supplementary Fig. 21). The width of microstructures can also be controlled by using micropillars with different widths (Supplementary Fig. 22).

### Stretchability and electronic performance

**Fig. 2:**
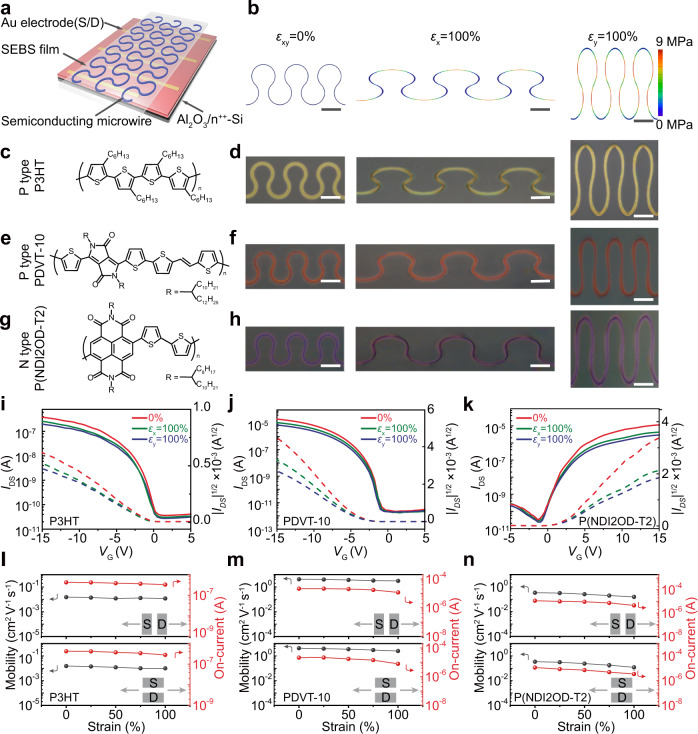
Stretchability of polymer curvilinear microstructures. **a** Scheme of the FET device based on curvilinear polymers microstructures. The electronic performance of curvilinear polymer microstructures was measured by the soft contact lamination method. **b** Finite element analysis of the deformable curvilinear P3HT microstructure arrays on SEBS substrate in the unstretched state (*ε*_xy_ = 0%) and under 100% strain parallel (*ε*_x_ = 100%) and perpendicular (*ε*_y_ = 100%) with the charge transport direction, respectively. Chemical structures of two typical P-type polymers **c** P3HT and **e** PDVT-10, and an N-type polymer (**g**) P(NDI2OD-T2). **d**, **f**, **h** Optical images of the deformable curvilinear microstructures based on polymers of (**c**), (**e**), and (**g**) on SEBS substrate in the unstretched state (left) and at 100% strain parallel (middle) and perpendicular (right) to the charge transport direction, respectively. **i**–**n** Transfer curves and the statistical diagrams of the on-current and mobility of these polymer OFETs at 0% strain and 100% strain parallel and perpendicular to the charge transport direction, respectively. Solid and dashed lines in (**i**)–(**k**) are *I*_DS_–*V*_GS_ and |*I*_DS_|^1/2^–*V*_GS_ curves of devices, respectively. The mobilities in (**l**)–(**n**) represent average values are obtained from five devices. All scale bars: 10 μm.

To evaluate the stretchability, the curvilinear polymer microstructures were firstly fabricated onto a rigid substrate followed by transferring to stretchable polystyrene-block-poly(ethylene-ran-butylene)-block-polystyrene (SEBS) elastomer (see Method and schematics in Fig. 2a, Supplementary Fig. 23). The stretchability was firstly estimated by observing the morphology of P3HT curvilinear microstructures under different applied strains (Supplementary Figs. 24–26). For the curvilinear microstructures with a central angle of 270^o^, no obvious cracks can be observed under 100% strain along both horizontal and vertical directions (Supplementary Fig. 26). The crack-free microstructures under 100% strain are also validated by AFM images (Supplementary Fig. 27). In contrast, microstructures with a central angle of 90^o^ and 180^o^ present limited stretchability (Supplementary Figs. 24 and 25). Spin-coated thin films and line-shaped microstructures experience serious damage under 100% strain (Supplementary Fig. 28). The limited stretchability is rationalized by the stress–strain curves, illustrating that the damage of a bulky P3HT belt occurs with strain below 40% (Supplementary Fig. 29). Finite element analysis of curvilinear microstructures under 100% strain presents the spatial distribution of stress (Fig. 2b). The stress can be effectively released by deformation of curvilinear microstructures, yielding calculated stress below 8.3 MPa under 100% strain, which is well below the damage stress of *ca*. 20 MPa (Supplementary Fig. 29c). These results demonstrate that the curvilinear microstructures endow exotic stretchability to the conjugated semiconducting polymers with limited intrinsic flexibility.

Electronic transport properties were measured by contacting with gold electrodes to form an OFET device with a bottom-gate bottom-contact configuration (Fig. 2a, see Method for details). The curvilinear microstructures were fabricated based on three representative semiconductors, including n-type P(NDI2OD-T2), p-type P3HT, and PDVT-10 (Fig. 2c, e, g). No crack and damage can be observed in these three polymer microstructures under 100% applied strain along horizontal and vertical directions (Fig. 2d, f, h), demonstrating considerable stretchability of these curvilinear microstructures. The electronic transport performances were measured to directly evaluate the stretchability of OFET devices based on curvilinear microstructures. By comparing the transfer curves of devices under different strains (Fig. i-k), the electronic transport is well preserved under 100% strain parallel and perpendicular to the channel direction. The primary mobility of PDVT-10 curvilinear microstructures is 4.3 cm^2^ V^−1^ s^−1^, which is high than 1.1 cm^2^ V^−1^ s^−1^ on spin-coated thin films (Supplementary Fig. 30). The fourfold higher mobility in curvilinear microstructures can be attributed to the improved crystallinity and ordered molecular packing, which has been demonstrated by GIWAXS characterizations (Fig. 1h, Supplementary Figs. 19 and 30). To avoid the possible deviations on the mobility evaluation introduced by the curvilinear semiconducting channel, more rigorous measurements of mobility were performed based on field-effect mobility of straight microwires and space charge limited current (SCLC) mobility of hole-only devices. The field-effect and SCLC mobilities are 4.2 ± 0.5 and 9.8 ± 0.3 cm^2^ V^−1^ s^−1^, respectively, which is well consistent with the field-effect mobility of 4.3 cm^2^ V^−1^ s^−1^ based on curvilinear channels (Supplementary Figs. 31 and 32). The high mobility of assembled curvilinear microwires motivated us to study the charge-transport behavior, which can be characterized by temperature-dependent field-effect mobility^38–40^. A negative temperature coefficient of the mobility can be observed at temperatures ranging from 210 to 300 K, which evidences the band-like transport behavior in these microwires (Supplementary Fig. 33).

Under 100% strain along and perpendicular to the channel direction, the mobilities of 2.6 and 2.1 cm^2^ V^−1^ s^−1^ can be realized in PDVT-10 curvilinear microstructures, respectively. For assembled straight PDVT-10 wires, primary mobility of 3.9 cm^2^ V^−1^ s^−1^ can be achieved, whereas no electronic signal can be measured under 100% strain and a 390-fold decrease of mobility occurs after the release of the strain (Supplementary Fig. 34), indicating serious damage of straight wires in the stretch process. For P3HT, PDVT-10, P(NDI2OD-T2) curvilinear microstructures, over 40% preservation of mobility can be achieved under 100% strain (Fig. 2l–n), indicating the robust stretchability of these curvilinear microstructures.

### Fully stretchable electronics based on curvilinear microstructures

**Fig. 3:**
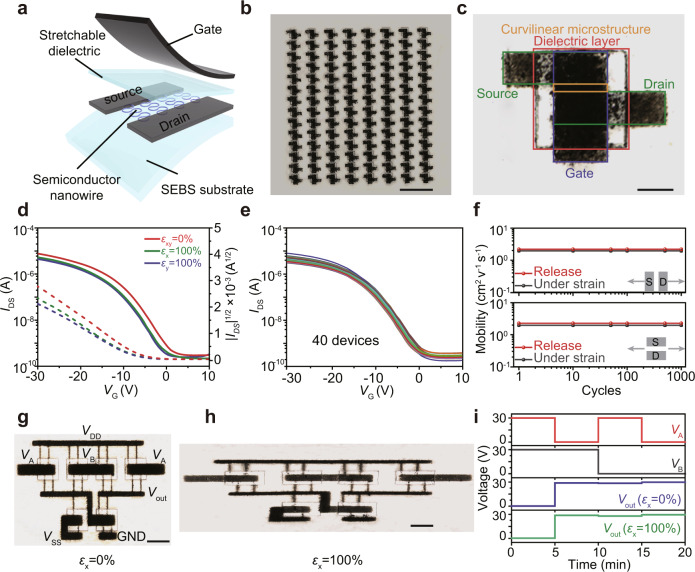
Electronic performance of fully stretchable OFET arrays. **a** Schematic illustration of a fully stretchable OFET constructed by curvilinear microstructure arrays as the semiconducting channel, SEBS as gate dielectrics, CNTs as the source, drain, and gate electrodes. **b** Optical image of 112 fully stretchable OFET arrays. **c** Zoom-in image of an OFET device with the channel length of 30 μm, the width of 90 μm, gate dielectric thickness of 1.2 μm, and capacitance of 1.5 nF cm^−2^. **d** Transfer curves of the fully stretchable PDVT-10 polymer OFET (*V*_D_ = −30 V) in the unstretched state and at 100% strain parallel and perpendicular to the charge transport direction. Solid lines and dashed lines are *I*_DS_–*V*_GS_ curves and |*I*_DS_|^1/2^–*V*_GS_ curves of these devices, respectively. **e** Transfer curves of 40 devices in the 112-transistor arrays at 0% strain. **f** Statistical mobilities of OFETs during up to 1000 stretch-release cycles at 50% strain parallel (*ε*_x_ = 50%) and perpendicular (*ε*_y_ = 50%) to the charge transport direction. Each point in (**f**) represents average values obtained from five OFET devices. **g**, **h** Optical images of stretchable NAND gate (**g**) in its initial state and (**h**) at 100% applied strain in the horizontal direction, where *V*_A_ and *V*_B_ are the input voltages at A and B terminals, respectively. **i** Output–input characteristic curves of the NAND gate at 0% and 100% strain. Scale bars; **b** 4 mm, **c** 400 μm, **g** 100 μm, **h** 100 μm.

The reliable stretchability of these curvilinear microstructures permits the construction of fully stretchable electronics. These flexible electronic devices with a top-gate top-contact configuration were fabricated by transferring PDVT-10 curvilinear structures onto SEBS elastomer followed by coating with SEBS and stretchable carbon nanotubes (CNTs) as flexible gate dielectrics and electrodes, respectively (Supplementary Figs. 23 and 36, see Method for detailed discussion). The optical micrographs demonstrate the successful transfer of curvilinear microstructures onto SEBS substrate and the AFM image illustrate the high quality of patterned SEBS dielectrics (Supplementary Fig. 36c). Totally, 112 fully stretchable OFETs can be fabricated onto SEBS substrate (Fig. 3b) and a zoom-in optical image presents the alignment of semiconducting curvilinear microstructures, CNT electrodes, and SEBS gate dielectrics (Fig. 3c). Figure 3d presents representative transfer curves of the champion device with primary carrier mobility of 2.1 cm^2^ V^−1^ s^−1^ and an on-off ratio of 10^4^. The electronic performance is well preserved under 100% strain parallel and perpendicular to the channel direction. The reproducibility of the electronic performances is examined by statistics of 40 devices, yielding average mobility of 1.5 cm^2^ V^−1^ s^−1^ based on this fully stretchable configuration (Fig. 3e). To evaluate the stretchability, the OFET device was measured under cyclic stretch and release with a maximum strain of 50%. The OFET presents 92% preservation of mobility after 1000 cycles (Fig. 3f, Supplementary Fig. 37), indicating the robustness of the stretchability. To evaluate the impacts of the transfer process on the contact and semiconductor–dielectric interface, we carried out the measurements of contact resistance and subthreshold slope. Contact resistance of 0.61 kΩ cm is determined by the transmission line method, which is not a constraint for carrier transport compared to the channel resistance (Supplementary Fig. 38). The interfacial trap density can be reflected by the subthreshold slope (see details in Supplementary Fig. 39), which is lower for SEBS dielectric compared to ALD Al_2_O_3_.

The scalable fabrication of curvilinear microstructures, dielectrics, and CNT electrodes also allows for the construction of large-area device arrays for wearable electronics. 2000 stretchable OFETs were fabricated onto SEBS substrate in an area of *ca*. 2 × 2 cm^2^ (Supplementary Fig. 40) and these device arrays are stable under 100% strain (Supplementary Fig. 41). These OFET arrays can serve as wearable electronic devices due to their fully stretchable nature (Supplementary Fig. 42). To demonstrate the reliability of stretchable devices in practical situations, various deformations, including twisting, pressing, and biaxial stretching, have been applied and no observable degradation indicates the robustness of these devices (Supplementary Fig. 43). Due to the scalable fabrication techniques, fully stretchable logic circuits can be constructed based on curvilinear microstructures. A NAND logic gate was fabricated by connecting six OFETs (Fig. 3g, Supplementary Fig. 44). By sweeping the two inputs *V*_A_ and *V*_B_, a low output voltage of near-zero can be observed only when the two inputs are at a high voltage of 30 V, indicating the NAND logic function (Fig. 3i). Stretchable semiconducting channels, gate dielectrics, and CNT electrodes enable logic function under 100% strain (Fig. 3h, i). Due to the curvilinear microstructures with long-range uniformity and high ordered molecular packing, scalable integration of stretchable electronic device arrays is realized based on semiconducting polymers. Compared to previous reports, our method by incorporating geometry engineering in the assembly of polymers provides a platform to overcome the trade-offs between electronic performance, stretchability, and scalability (Supplementary Table 1).

### Wafer-scale uniformity and deterministic patterning

**Fig. 4:**
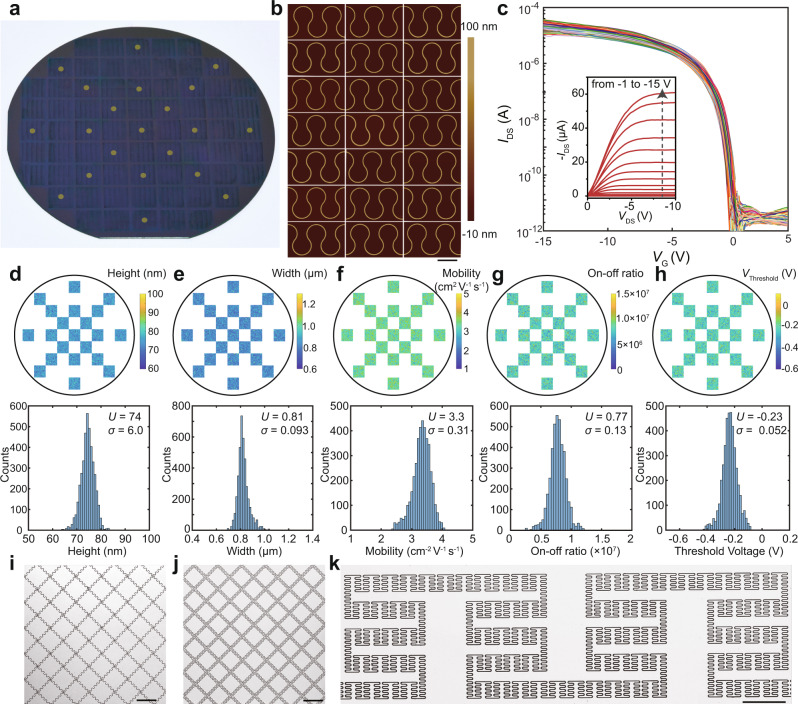
Wafer-scale homogeneity of curvilinear polymer microstructures. **a** Curvilinear polymer microstructure arrays on the 4-in. silicon wafer deposited with 50 nm Al_2_O_3_ as dielectrics. Twenty-one sites are labeled for the statistics of size and electronic performance across the wafer. **b** Representative AFM images of curvilinear microstructures collected from different labeled sites in (**a**). **c** Transfer curves and output curves of PDVT-10 OFET arrays measured across the 4-in. wafer. Statistics of height (**d**), width (**e**), mobility (**f**), on–off ratio (**g**), threshold voltage (**h**) at 21 sites across the wafer. Colormaps (top panel) presents the spatial distribution of sizes and electronic performances, which is measured from 21 labeled sites with 196 points in each site. The bottom panels show the statistics of sizes and electronic performances with calculated mean values (*U*) and standard deviations (*σ*). **i**, **j** Optical micrographs of 2D meshes constructed by curvilinear polymer microstructures. **k** Optical microscope image of multi-order hierarchical fractal patterns composed of curvilinear polymer microstructures. Scale bars: **b** 10 μm, **i**, **j** 50 μm, **k** 200 μm.

Given that the sequential dewetting and efficient trapping of liquid in microreservoirs permit the fabrication of long-range uniform curvilinear structures, we sought to demonstrate the wafer-scale fabrication of stretchable polymer microstructures. Figure 4a shows curvilinear structures fabricated onto a 4-in. silicon wafer. To evaluate the long-range uniformity, we measured both geometric and electronic-transport figures of merits across 21 different positions on this wafer. Typical AFM images extracted from these 21 different positions show uniform width and height of curvilinear microstructures at long range (Fig. 4b). FETs were fabricated based on these curvilinear microstructures using a top-contact bottom-gate configuration and transfer curves extracted from *ca*. 4000 devices from these 21 positions illustrate the narrow distribution of on and off currents (Fig. 4c). Based on these devices in different positions, figures of merit, including mobility, on–off ratio, and threshold voltage were extracted. Spatial distribution profiles of height, width, mobility, on–off ratio, and threshold voltage illustrates long-range uniformity and reproducibility of both geometric and electronic parameters across the 4-in. wafer (Fig. 4d–h). By statistics of these parameters, an average height of 74.0 ± 6.0 nm, the width of 0.81 ± 0.09 μm, mobility of 3.3 ± 0.3 cm^2^ V^−1^ s^−1^, on–off ratio of (7.7 ± 1.3) × 10^6^, and the threshold voltage of −0.23 ± 0.05 V can be extracted. The narrow distributions of these parameters indicate the high uniformity of these curvilinear microstructures on a wafer-scale.

This technique can be further extended to fabricate 2D curvilinear networks by rational design of templates. For instance, 2D meshes constructed by curvilinear wires with central angles of 180^o^ and 270^o^ can be successfully fabricated (Fig. 4i, j, Supplementary Fig. 45). This sequential dewetting strategy can be further employed in the fabrication of multi-order hierarchical fractal patterns (Fig. 4k). The sequential dewetting process is driven by a capillary gradient, yielding a homogeneous distribution of liquid into capillary bridges (Supplementary Fig. 46).

## Discussion

In conclusion, high-quality stretchable curvilinear microstructures of semiconducting polymers have been realized at wafer scale by steering capillary gradient. Ordered molecular packing and designable curvilinear microstructures are demonstrated based on three different polymers, leading to the high mobility of 4.3 cm^2^ V^−1^ s^−1^ and well-preserved electronic performance under 100% strain and less than 8% degradation of performance after 1000 stretch-release cycles under 50% strain. This method also boosts the integration of fully stretchable FET and logic gate arrays. Controllable dewetting dynamics and efficient trapping of liquid in microreservoirs enable long-range uniformity of curvilinear microstructures, which is reflected by narrow distributions of height, width, mobility, on–off ratio, and threshold voltage at wafer scale. It is anticipated that this solution-processing approach benefits the large-scale and high-throughput integration of wearable electronic devices, which is not restricted by molecular design.

## Methods

### Assembly of curvilinear semiconductor microstructure arrays

P-type semiconducting polymers P3HT (*M*_n_: 100 kDa) and PDVT-10 (*M*_n_: 65 kDa), and N-type semiconducting polymer P(NDI2OD-T2) (PolyeraActivInk^™^ N2200, *M*_n_: 63.8 kDa) were used in this study. The polymers were dissolved in chloroform at a concentration of 5 mg mL^−^^1^. A 10 µL droplet of the polymer solution was dropped onto the curvilinear template and covered by the target substrate, yielding a capillary-bridge assembly system. The assembly system was kept at room temperature for 24 h to evaporate the organic solvents. After the total evaporation of solvents, the semiconductor microstructure arrays were generated onto the target substrate.

We developed two methods to transfer curvilinear semiconductor microstructures to the stretchable SEBS substrate: (1) The curvilinear semiconductor microstructures were firstly assembled on the SiO_2_ surface by using the capillary-bridge assembly. Next, SEBS 1221 with a concentration of 300 mg mL^−^^1^ was spin-coated on the SiO_2_ surface at 1000 rpm for 60 s. The curvilinear semiconductor microstructures embedded in SEBS substrates were released from the SiO_2_ surface by dipping into ethanol solution. (2) The 10 nm Cr/100 nm Cu were deposited on the silicon surface as a sacrificial layer by the e-beam evaporation. The curvilinear semiconductor microstructures were firstly assembled on the Cu surface using capillary bridge lithography. Next, SEBS 1221 with a concentration of 300 mg mL^−1^ was spin-coated on the copper surface at 1000 rpm for 60 s. The curvilinear semiconductor arrays were transferred from the sacrificial copper film to SEBS substrates using FeCl_3_ solution. Compared with the first method, the second method is appropriate for transferring polymer microstructures, which strongly adhere to the SiO_2_ substrate.

### Morphological and crystallographic characterizations

Optical and fluorescence microscope images of the dewetting process and as-formed polymer microstructures were obtained by an optical microscope (Vision Engineering Co., UK). The structures of the curvilinear micropillar templates and the morphology of curvilinear polymer microstructure arrays were investigated by the SEM (Hitachi, S-4800, Japan). The AFM images and surface roughness data were measured by the Nanoscope IIIa instrument (Bruker, ICON2-SYS, Germany). The GIWAXS data were obtained at 1W1A Diffuse X-ray Scattering Station, Beijing Synchrotron Radiation Facility (BSRF-1W1A) with an incidence angle of 0.2°. The tensile strength of P3HT belts (1 mm × 6 μm × 20 mm) and SEBS belts (1 mm × 20 μm × 20 mm) was measured by dynamic mechanical analysis (DMA, Q800, TA Instrument, USA) with a stretching rate of 0.2 mm min^−1^.

### Observation and simulation of the dewetting process

To in situ observe the dewetting process in the gap between adjacent curvilinear micropillars, a polymer, poly(9,9-dioctylfluorene-co-benzothiadiazole) (F8BT) with green fluorescence, was added into the solution. The dewetting process was observed by a fluorescent microscope (Vision Engineering Co., UK). COMSOL Multiphysics was used to simulate the dewetting behavior. The contact angle the silicon pillar is *ca*. 0^o^. The surface tension is set as 28.8 mN m^−1^. The radius of the central arc pillar is 10 μm. The defined minimum spacing between two pillars is 15 μm.

### Finite element analysis

COMSOL Multiphysics was used to study the mechanical response of the curvilinear semiconductor microstructures on the stretchable SEBS substrate. In our simulation, a curvilinear P3HT microstructure with the exposed top surface was modeled by four-node composite shell elements, which were embedded in the SEBS 1052 substrate and have an arc angle of 270^o^, arc radius of 5 µm, the width of 600 nm, and thickness of 400 nm. Displacement boundary conditions were applied to both edges of the substrate to produce uniaxial tension of the substrates. The material parameters of SEBS and P3HT are *E*_SEBS_ = 1 MPa and *ν*SEBS = 0.49, and *E*_P3HT_ = 28 MPa and *ν*_P3HT_ = 0.35, respectively. Here, *E* is the elastic modulus and *ν* is the Poisson’s ratio.

### The electrical measurements using the soft contact lamination method

The high *k* gate dielectric layer Al_2_O_3_ (50 nm) was deposited on the surface of highly doped Si by the atomic-layer-deposition (ALD) method. The electrical performance of stretchable curvilinear semiconductor microstructures was measured through the bottom-gate bottom-contact OFET by the soft contact lamination: Al_2_O_3_ film as the high *k* gate dielectric, highly doped Si as the gate electrode, and Au film on the top as the source and drain electrodes.

### Fabrication and measurement of devices

The fully deformable OFET device was fabricated as follows: (1) The curvilinear semiconductor microstructures were transferred to SEBS 1221 substrate. (2) P3-SWCNT was dispersed in 2-propanol at a concentration of 5 mg mL^−1^. The CNT solution was spray-coated onto curvilinear microstructure arrays as the source and drain electrodes by the pneumatic spray gun with a shadow mask. (3) A dextran solution (*M*_n_: 5000, 40 wt% in water) was spin-coated on a plasma-treated quartz glass slide at 1500 rpm for 20 s. The glass slide was then baked at 80 °C for 30 min. Then, the SEBS (H1052) solution (10 wt% in toluene) was spin-coated onto the sample at 1000 rpm for 60 s as a stretchable dielectric layer with a thickness of *ca*. 1.2 μm. The surface of the SEBS dielectric layer was modified by OTS to increase the dielectric performance. The dielectric layer is patterned via direct laser writing through a nanosecond, pulsed UV laser beam with the computer-assisted design. (4) The quartz glass substrates with the SEBS dielectric layer were laminated onto stretchable SEBS (H1221) substrate with curvilinear semiconductor microstructures with precise alignment. Then, the dielectric layer on the substrate was transferred onto the stretchable substrate by immersing the entire device in water to dissolve the sacrificial dextran layer. The device was thermally annealed at 80 °C in a vacuum for 4 h to enhance the contact between the dielectric layer and the organic semiconductor layer. (5) Finally, a CNT layer was spray-coated through a metal shadow mask onto the dielectric layer as the gate electrode.

All of the electrical characteristics of the OFETs were measured with the Keithley 4200A-SCS characterization system in an ambient environment at room temperature. The dielectric constants of SEBS 1052 were obtained by the capacitance measurement through an Agilent precision LCR meter E4980A. The carrier mobility in the saturation regime at gate voltage (*V*_G_) of −15 V could be calculated by

2 $$\mu =\frac{2L}{W{C}_{{{{{{\rm{i}}}}}}}}{\left(\frac{\partial \sqrt{|{I}_{{{{{{\rm{DS}}}}}}}|}}{\partial {V}_{{{{{{\rm{G}}}}}}}}\right)}^{2}$$

where *μ* is the carrier mobility, *L* is defined as the channel length, *W* is the channel width, *C*_i_ is the capacitance of the gate dielectric layer per unit area and *I*_DS_ is the current between the drain and source electrodes.

## Supplementary information

Supplementary Information

Description of Additional Supplementary Files

Supplementary Movie 1

## Data Availability

The data that support the findings of this study are presented in the paper and supplementary information. Source data are available from the corresponding author upon request.
