# Supplementary material for: Wafer-scale integration of stretchable semiconducting polymer microstructures via capillary gradient
Source: Nat Commun. 2021 Dec 2;12:7038. doi: 10.1038/s41467-021-27370-w (PMC8640044; doi:10.1038/s41467-021-27370-w)
Supplement: Supplementary file 1 — Supplementary Information [file 41467_2021_27370_MOESM1_ESM.pdf]

Supplementary Information

# Wafer-scale integration of stretchable semiconducting polymer microstructures via capillary gradient

Yuchen Qiu[1, 2, 8], Bo Zhang[3, 8], Junchuan Yang[1, *], Hanfei Gao[1], Shuang Li[4], Le Wang[5], Penghua Wu[1], Yewang Su[4], Yan Zhao[6, *], Jiangang Feng[7, *], Lei Jiang[1], Yuchen Wu[1]

[1] Key Laboratory of Bio-inspired Materials and Interfacial Science, Technical Institute of Physics and Chemistry, Chinese Academy of Sciences, Beijing 100190, P. R. China

[2] College of Chemistry, Jilin University, Changchun 130012, P. R. China

[3] Beijing Key Laboratory of Lightweight Multi-Functional Composite Materials and Structures, Institute of Advanced Structure Technology, Beijing Institute of Technology, Beijing 100081, People's Republic of China

[4] State Key Laboratory of Nonlinear Mechanics, Institute of Mechanics, Chinese Academy of Sciences, 100190 Beijing, P. R. China

[5] Department of Biomedical Engineering, Southern University of Science and Technology, Shenzhen, Guangdong 518055, P. R. China

[6] Department of Materials Science, Fudan University, Shanghai 200433, P. R. China

[7] Department of Chemical and Biomolecular Engineering, National University of Singapore, Singapore 117585, Singapore

[8] These authors contributed equally: Yuchen Qiu, Bo Zhang

*e-mail: yangjc@mail.ipc.ac.cn; zhaoy@fudan.edu.cn; j.feng@nus.edu.sg

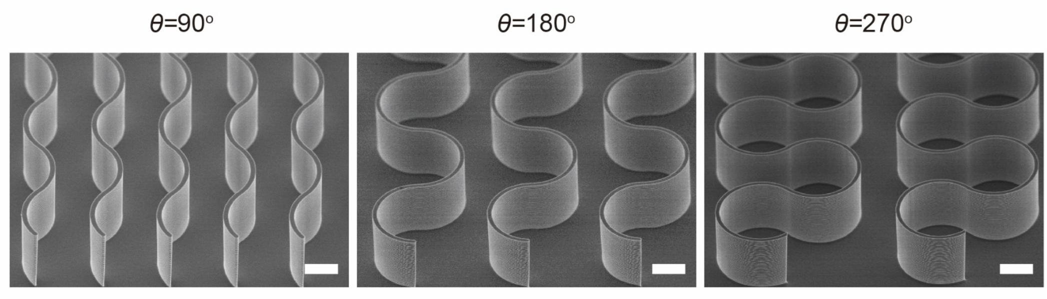


**Supplementary Fig. 1 | SEM images of curvilinear micropillar templates with central angle *θ* of 90°, 180° and 270°.** All scale bars, 10 μm.

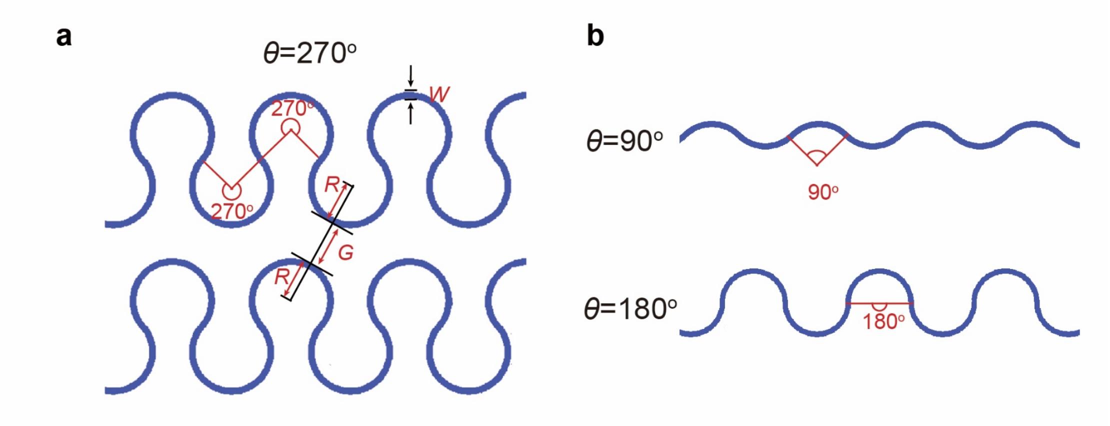


**Supplementary Fig. 2 | Geometrical parameters of curvilinear micropillars,** including the central angle (*θ*), arc radius (*R*), minimum gap distance (*G*), width (*W*) of micropillars. Schemes of micropillars with the *θ* of **a**, 270°, **b**, 90° and 180°.

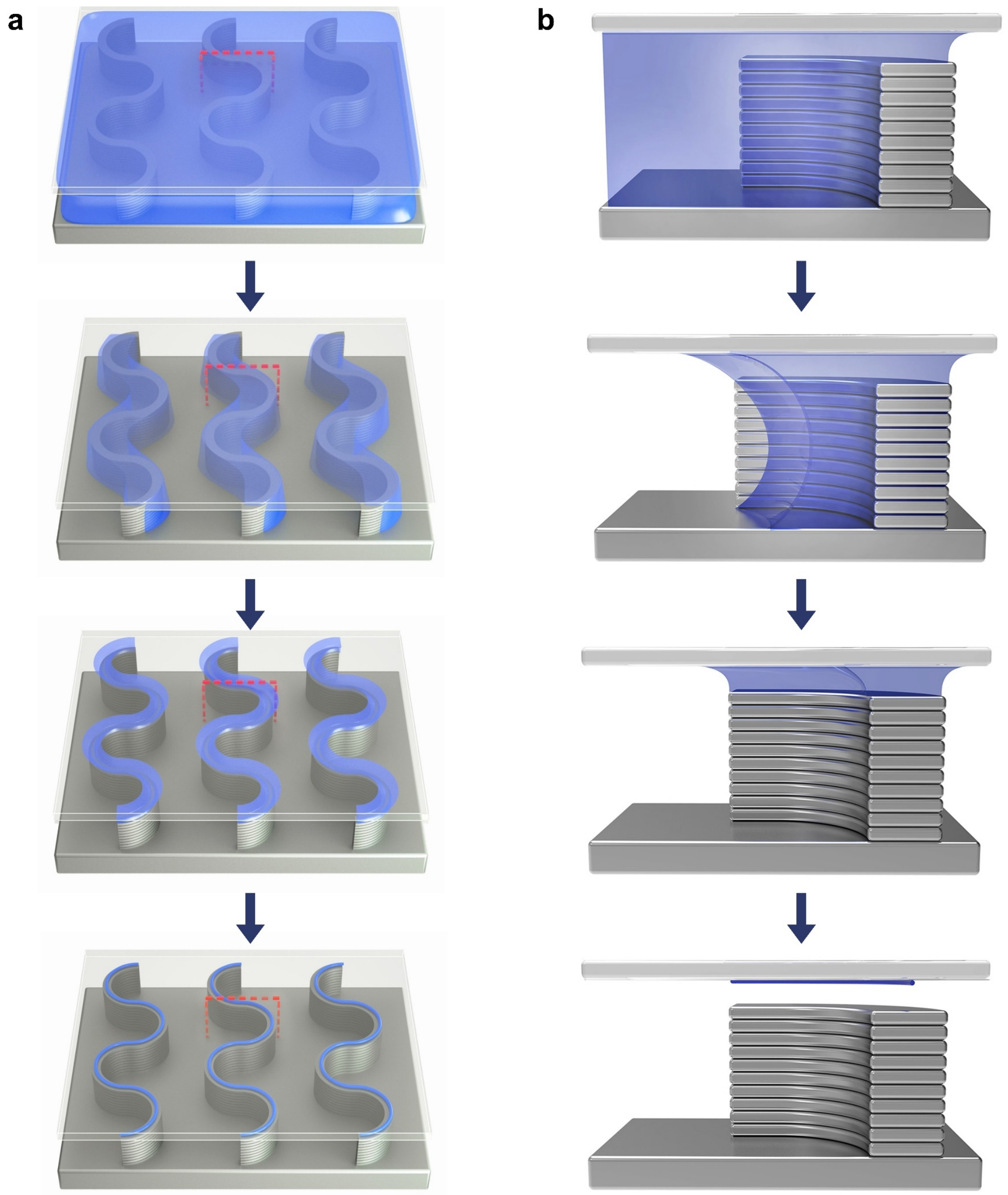


**Supplementary Fig. 3 | Schematic illustrations of fabrication process and assembly mechanism. a,** Schematic illustrations of the assembly process of curvilinear organic semiconductor microstructure arrays via the capillary-bridge lithography. **b,** Corresponding cross-sectional view schematic illustrations of the marked region in **a** during the dewetting process.

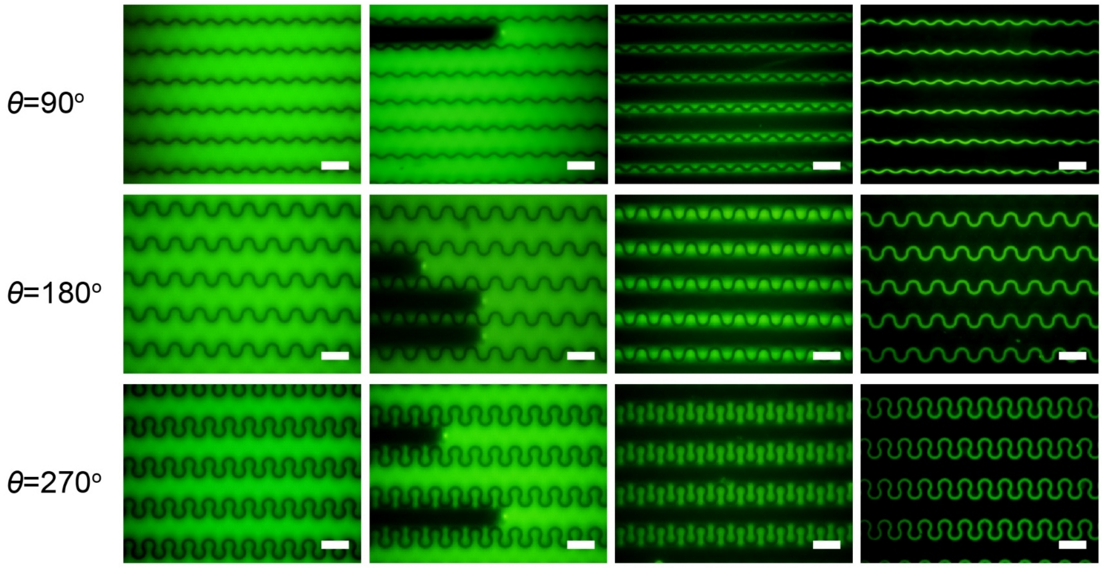


**Supplementary Fig. 4 | Fluorescence microscope observation of the dewetting process (*R* = 5 μm).** Dewetting process illustrated by fluorescence micrographs in micropillars with the arc radius of *R* = 5 μm, minimum inter-pillar distance of *G* = 15 μm and central angle $\theta$ of 90°, 180° and 270°. All scale bars, 20 μm.

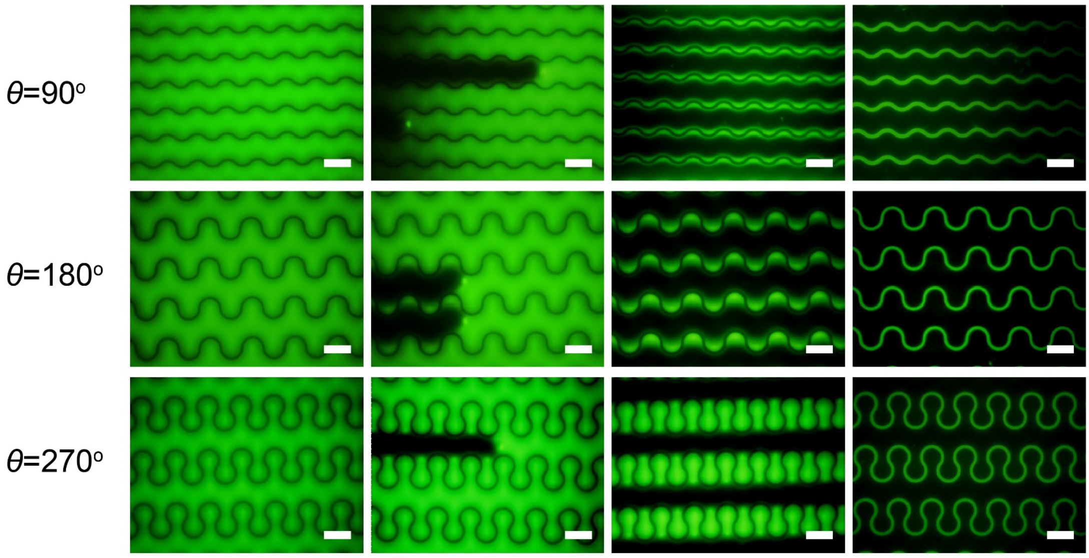


**Supplementary Fig. 5 | Fluorescence microscope observation of the dewetting process (*R* = 10 μm).** Dewetting process illustrated by fluorescence micrographs in micropillars with the arc radius of *R* = 10 μm, minimum inter-pillar distance of *G* = 15 μm and central angle $\theta$ of **a** 90°, **b** 180°, **c** 270°. All scale bars, 20 μm.

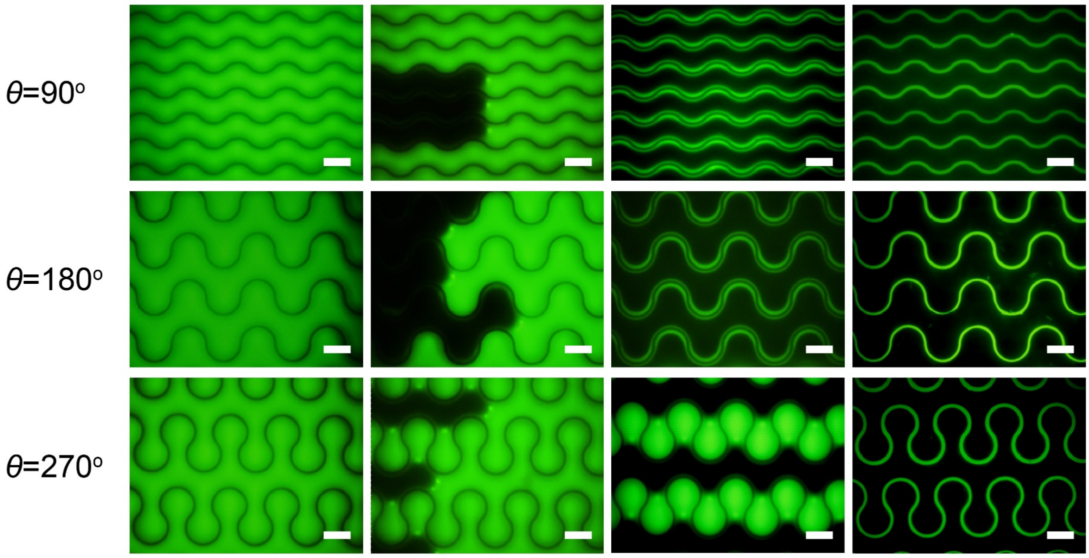


**Supplementary Fig. 6 | Fluorescence microscope observation of the dewetting process (*R* = 15 μm).** Dewetting process illustrated by fluorescence micrographs in micropillars with the arc radius of *R* = 15 μm, minimum inter-pillar distance of *G* = 15 μm and central angle *θ* of **a** 90°, **b** 180°, **c** 270°. All scale bars, 20 μm.

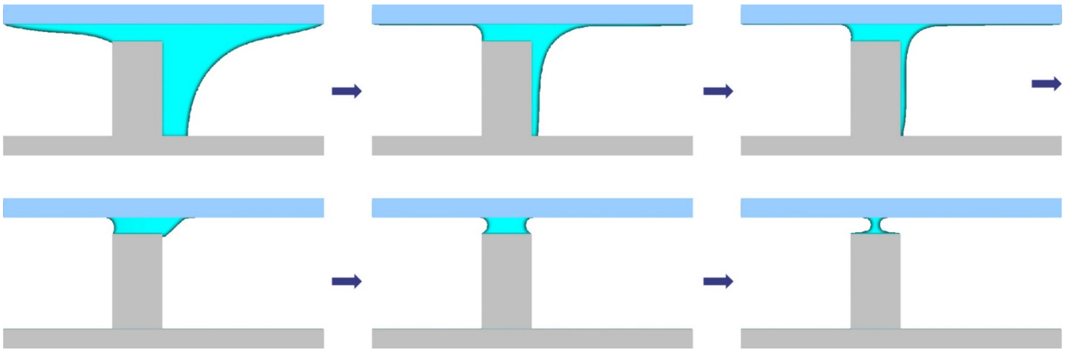

**Supplementary Fig. 7 | Fluid dynamics simulation of the dewetting process via the Lattice Boltzmann method.** The LBM simulation illustrates the directional dewetting process of the sandwiched assembly system from microreservoirs to capillary bridges, which is constructed by a micropillar template, the liquid layer and a flat substrate.

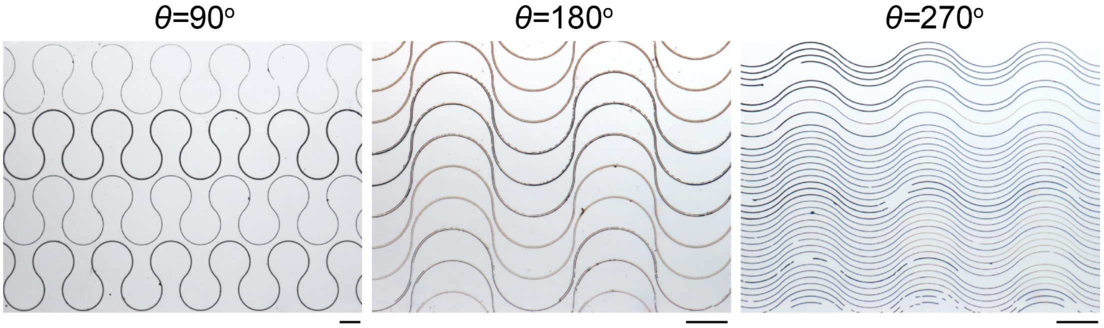


**Supplementary Fig. 8 | Optical microscope images of curvilinear semiconductor microstructure arrays assembled in the system without trapping of liquid in microreservoirs.** Curvilinear microstructure arrays with a broad size distribution assembled by the micropillars with central angles of $\theta$ = 90°, 180° and 270°, radius of $R$ = 100 μm and mimum gap distance $G$ = 10 μm. All scale bars, 100 μm.

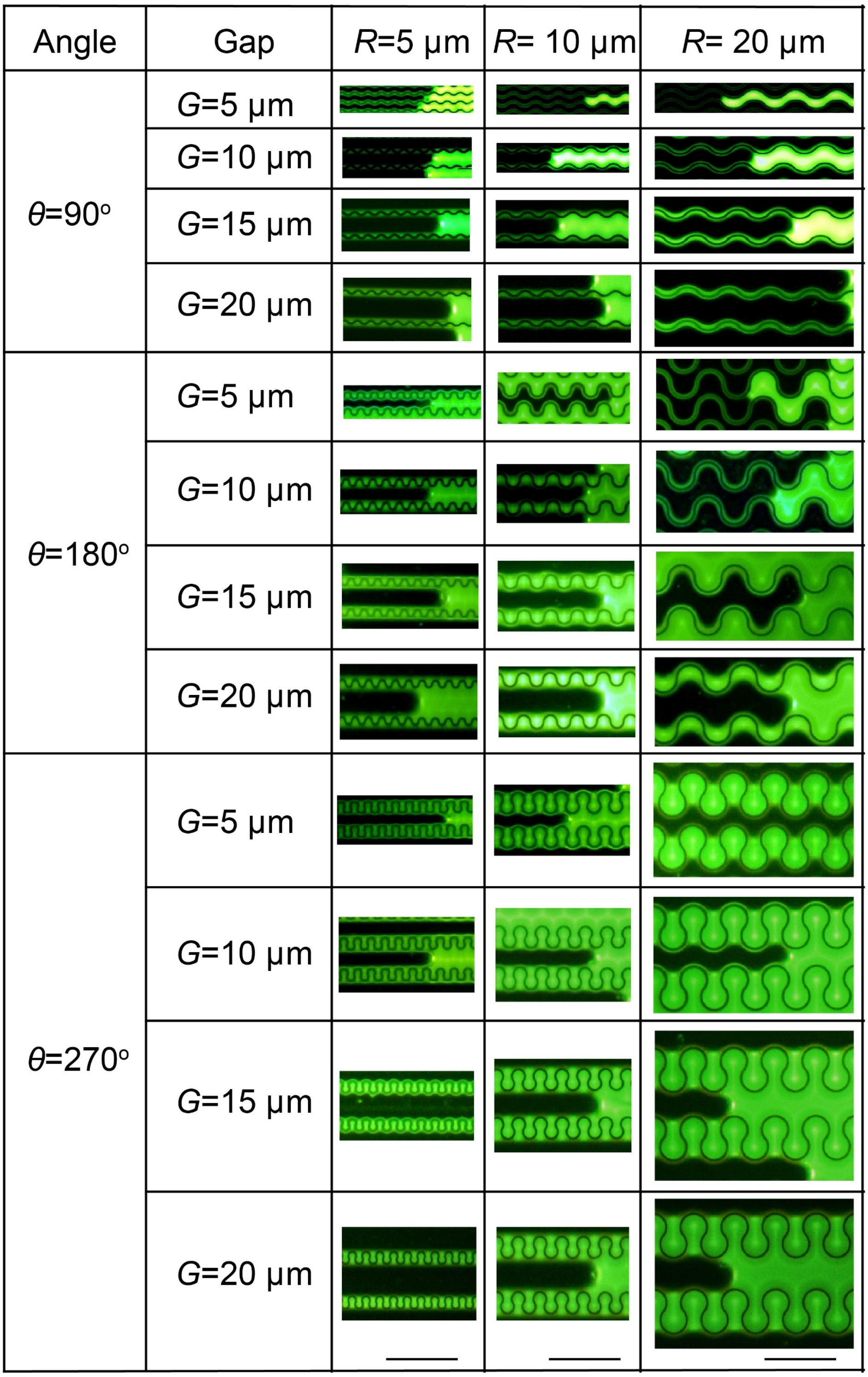


**Supplementary Fig. 9 | Dewetting behavior of precursor solutions in assembly system constructed by curvilinear micropillar templates with various geometries.** Representative fluorescence micrographs of the dewetting process with micropillar templates of $R$ = 5, 10 and 20 μm, $G$ = 5, 10, 15 and 20 μm and $\theta$ = 90°, 180° and 270°. All scale bars, 100 μm.

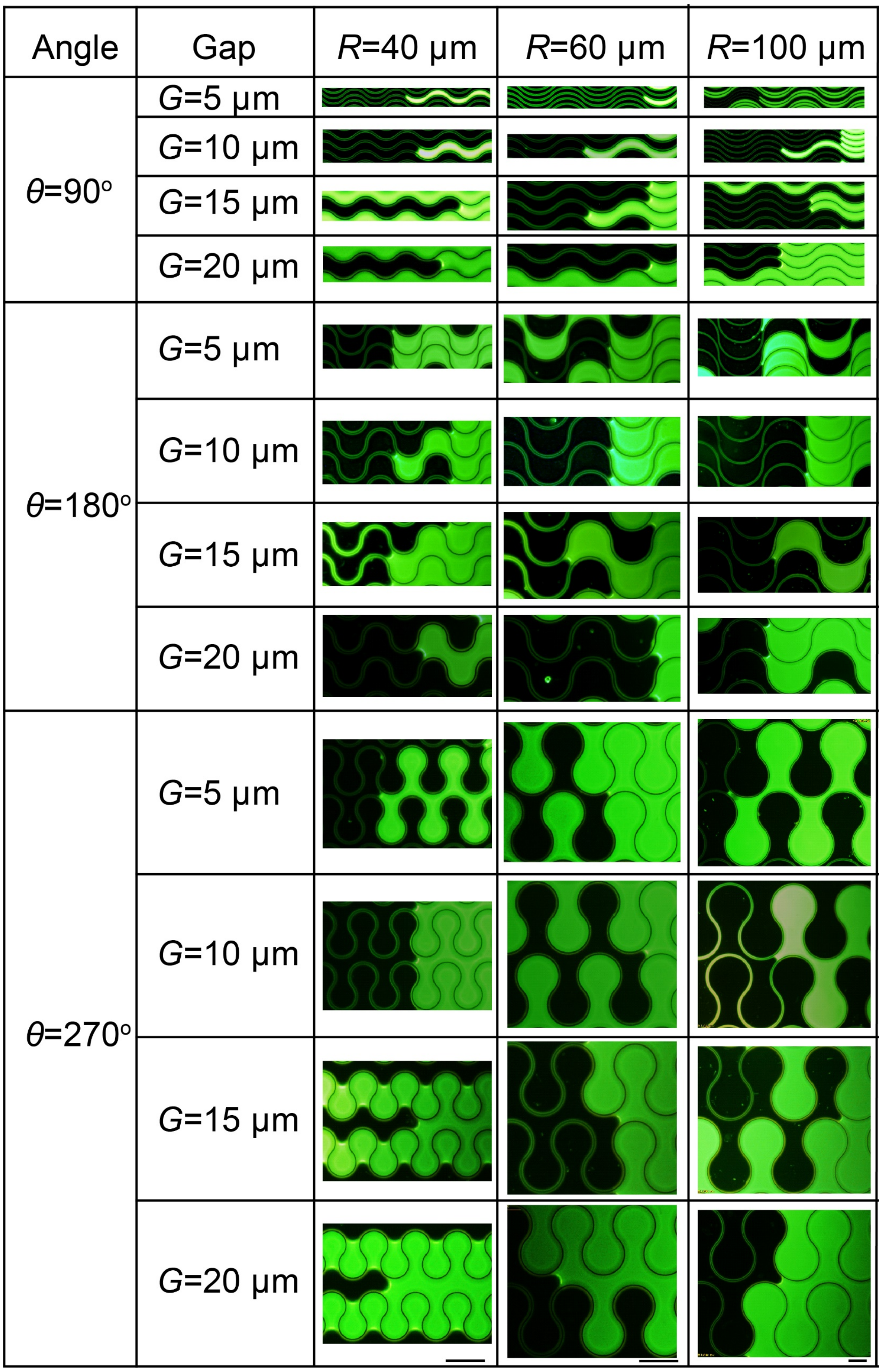


**Supplementary Fig. 10 | Dewetting behavior of precursor solutions in assembly system constructed by curvilinear micropillar templates with various geometries.** Representative fluorescence micrographs of the dewetting process with micropillar templates of $R$ = 40, 60 and 100 μm, $G$ = 5, 10, 15 and 20 μm and $\theta$ = 90°, 180° and 270°. All scale bars, 100 μm.

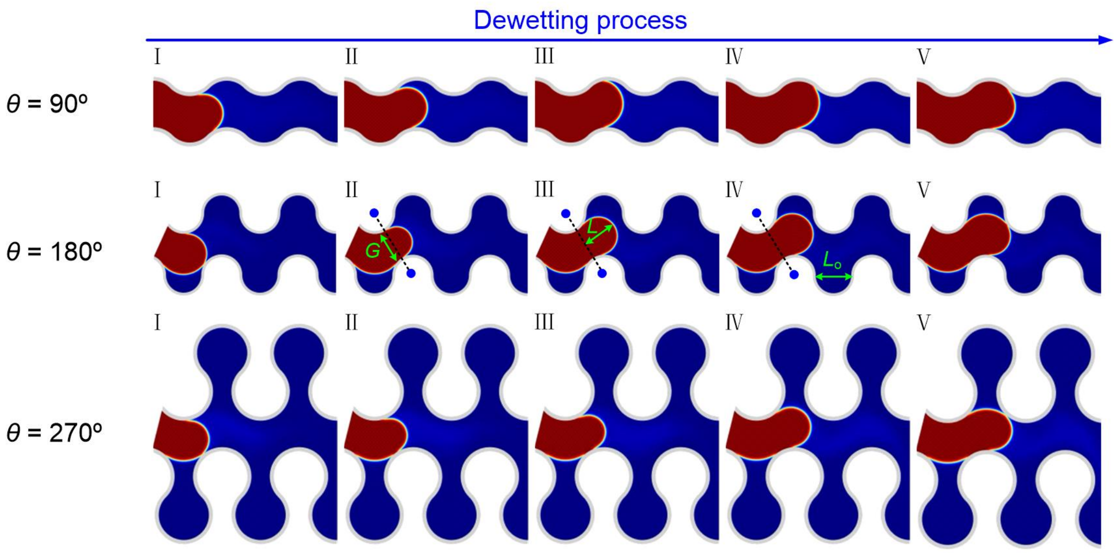


**Supplementary Fig. 11 | Fluid dynamics simulations of the dewetting process in the inter-pillar gap.** *L* is the length of liquid front. $L_o$ is the length of curvilinear opening. *G* is the minimum distance of the inter-pillar gap.

**Supplementary Note 1. Mechanism of liquid trapping in microreservoirs**

We performed a theoretical analysis to elucidate the mechanism of liquid trapping in microreservoirs. The model we built is shown in Supplementary Fig. 11, where blue and red blocks refer to the gas phase and liquid phase, respectively. We analyzed the dewetting process through fluid dynamics simulations. It should be noticed that the tangent slope of micropillar's convex changes from positive to negative (micropillar's concave possesses the opposite characteristic). If the shape of liquid-gas interface remains unchanged after a *dx* movement, the contact angle decreases (for the upper pillar) / increases (for the subjacent pillar) due to the convex shape. The slip process takes place when the contact angle is smaller than the receding contact angle or stick process take place. If the contact angle is smaller than the receding contact angle, the slip process takes place; otherwise stick process takes place. The phenomenon of slip process at one side and stick-slip process at the other side is always observed for the upper/subjacent convex pillar in the simulation (Supplementary Fig. 11, Supplementary Movie 1, 2 and 3). The stick process simultaneously takes place on both sides when the three-phase contact line moves to the connection of arc centers (180º and 270º (II)). The three-phase contact line remains motionless, but the liquid front keeps moving forward (180º and 270º (III-IV)). The simulation results show that the liquid-gas interface touches the next arc if the length of liquid front ($L$) is larger than the length of curvilinear opening ($L_o$), which means the receding liquid could not be trapped in the inner arc (180º and 270º (V)). If the arc angle of the micropillar is 90º, the liquid recedes smoothly along the arc (I-V). Thus, the threshold value for liquid trapping is $L_o$. The expressions of $L$ and $L_o$ are as follows:

$$L = \frac{G - G\sin\theta_Y}{2\cos\theta_Y} \tag{1}$$

$$L_o = \begin{cases} 2R\sin\frac{\theta}{2}(0 < \theta \le \pi) \\ 4R\cos\frac{\theta-\pi}{2} - 2R(\pi < \theta < 2\pi) \end{cases} \tag{2}$$

where $G$ is the minimum inter-pillar width, $\theta$ is the arc angle of the micropillar, $\theta_Y$ is

the Young's contact angle, and $R$ is the radius of arc.

Setting fixed $R$ and $L = L_o$, the threshold gap distance ($G_t$) for liquid trapping in microreservoirs can be calculated by

$$G_t = \begin{cases} 4R\sin\frac{\theta}{2}\frac{\cos\theta_Y}{1-\sin\theta_Y}(0<\theta\leq\pi) \\ 4(2R\cos\frac{\theta-\pi}{2}-R)\frac{\cos\theta_Y}{1-\sin\theta_Y}(\pi<\theta<2\pi) \end{cases} \quad (3)$$

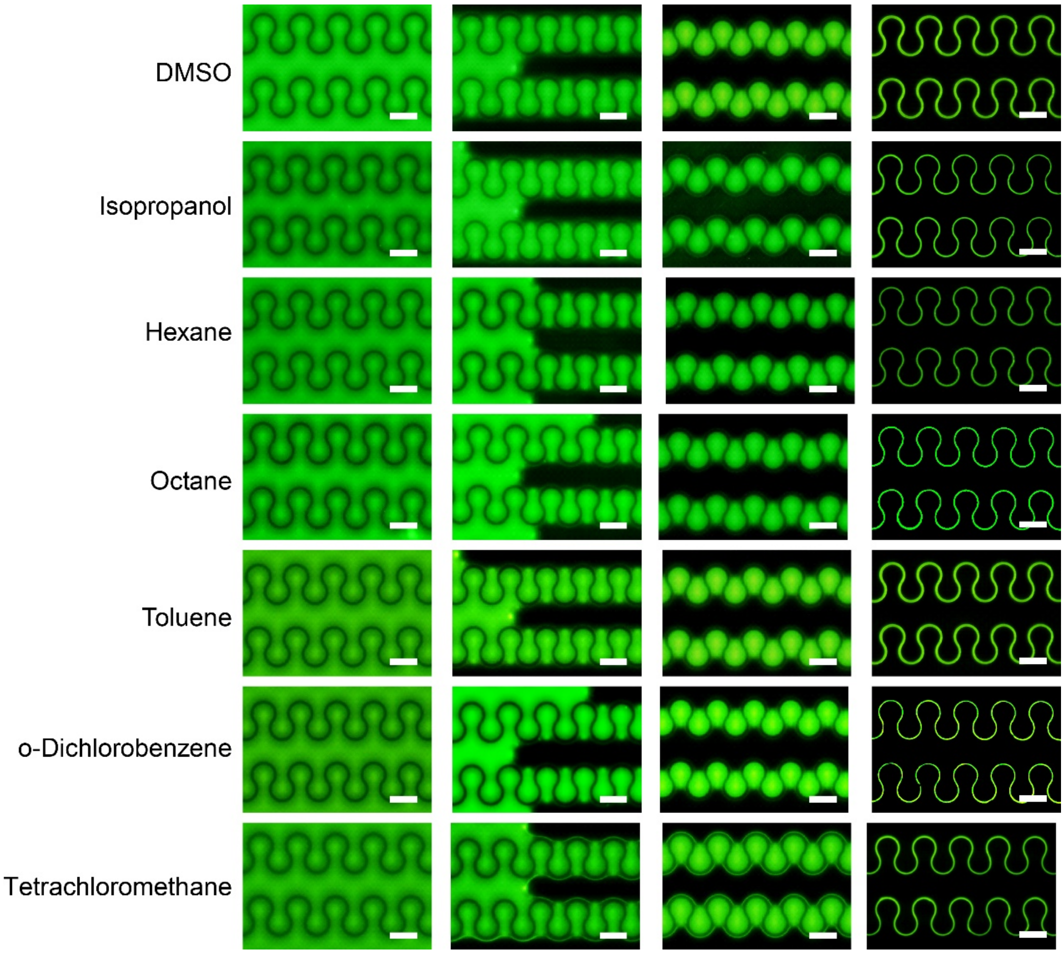


**Supplementary Fig. 12 | Assembly of curvilinear polymer microstructures based on different solvents.** Dewetting and assembly process of polymer solutions with solvents of DMSO ($\eta$ = 2.24 Pa·s), isopropanal ($\eta$ = 4.13 Pa·s), hexane ($\eta$ = 0.33 Pa·s), octane ($\eta$ = 0.53 Pa·s), toluene ($\eta$ = 0.59 Pa·s), o-dichlorobenzene ($\eta$ = 2.31 Pa·s), and terachloromethane ($\eta$ = 0.97 Pa·s), where $\eta$ is the viscosity of solvent determined by rheometer. Dewetting process illustrated by fluorescence micrographs in micropillars with the arc radius of $R$ = 15 μm, minimum inter-pillar distance of $G$ = 30 μm and central angle $\theta$ =270°. All scale bars, 30 μm.

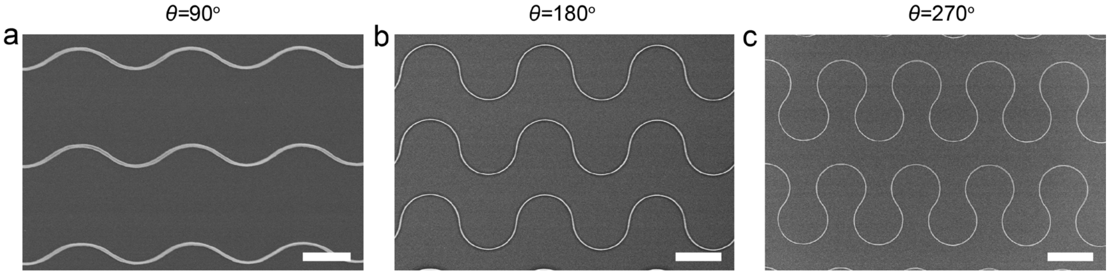


**Supplementary Fig. 13 | SEM images of curvilinear P3HT microstructure arrays with central angle of $\theta$ = 90°, 180° and 270°.** Scale bars: **a** 10 μm, **b** 20 μm, **c** 20 μm.

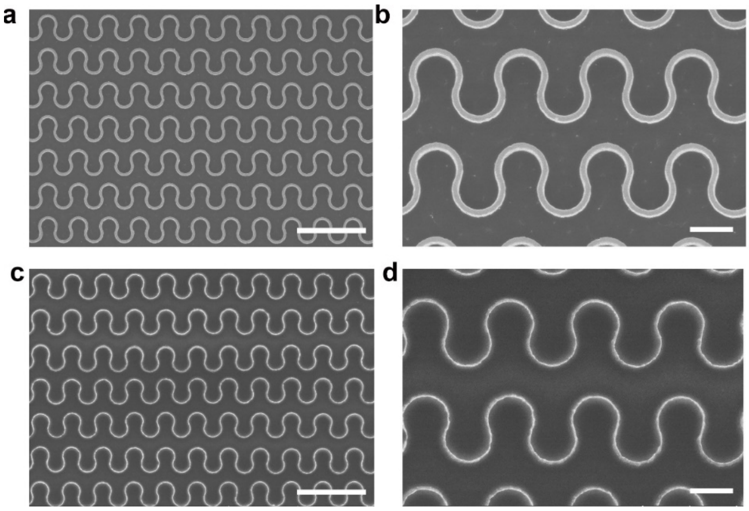


**Supplementary Fig. 14 | The nanowires fabricated by the capillary-gradient-mediated assembly method. a,** Low-magnification and **b**, zoom-in SEM image of curvilinear micropillar templates fabricated by electron-beam lithography and reactive-ion etching with radius $R$ of 1.2 μm, central angle $\theta$ of 270° and width $W$ of 350 nm. **c,** Low-magnification and **d**, zoom-in SEM image of curvilinear P3HT nanowires of 150 ± 40 nm in width and 2 μm in arc diameter. Scale bars: **a**, 10 μm, **b**, 2 μm.

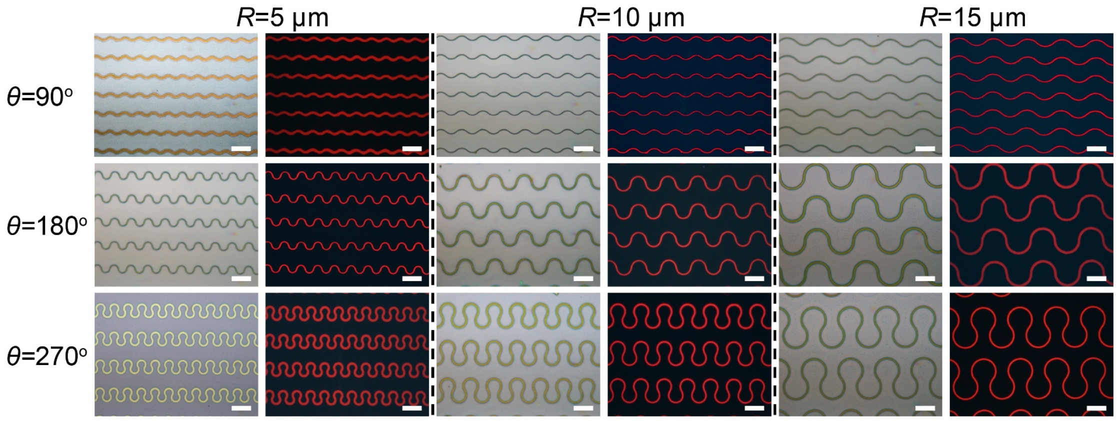


**Supplementary Fig. 15 | Bright-field optical and fluorescence microscope images of curvilinear P3HT microstructure array.** P3HT microstructure arrays with central arc angle of $\theta$ = 90°, 180° and 270°, and radius of $R$ = 5, 10 and 15 μm. All scale bars, 20 μm.

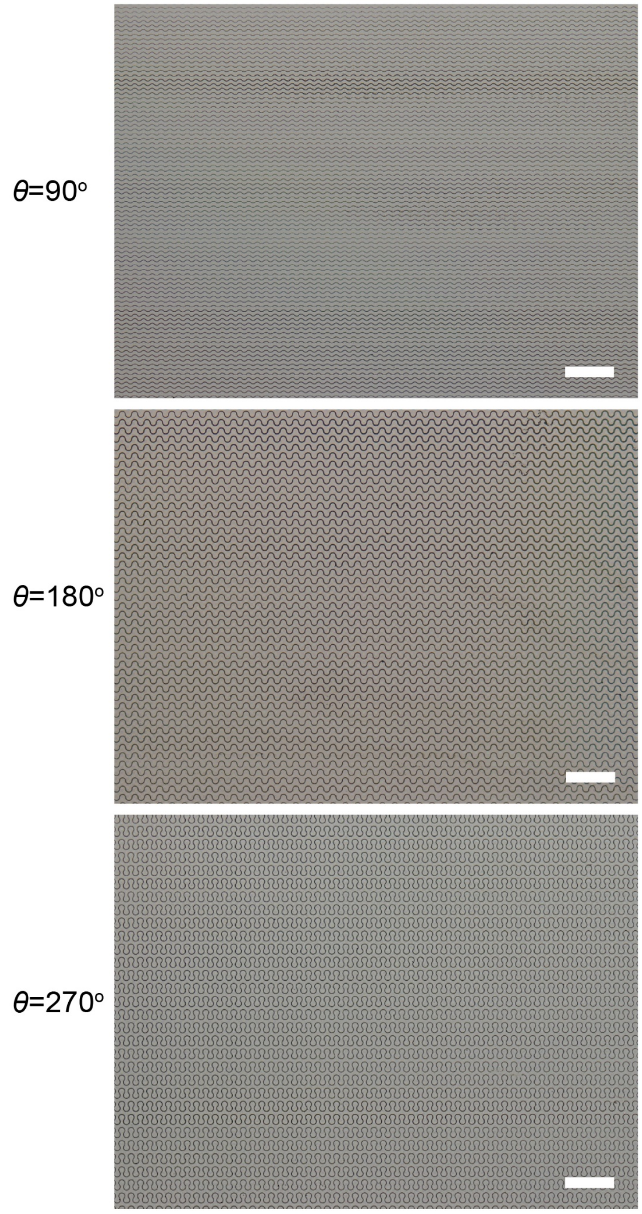


**Supplementary Fig. 16 | Fabrication of large-area curvilinear microstructure arrays with homogeneous morphology.** Large-area optical microscope images of curvilinear conjugated polymer microstructure arrays with central arc angle of $\theta$ = 90°, 180° and 270°. All scale bars, 200 μm.

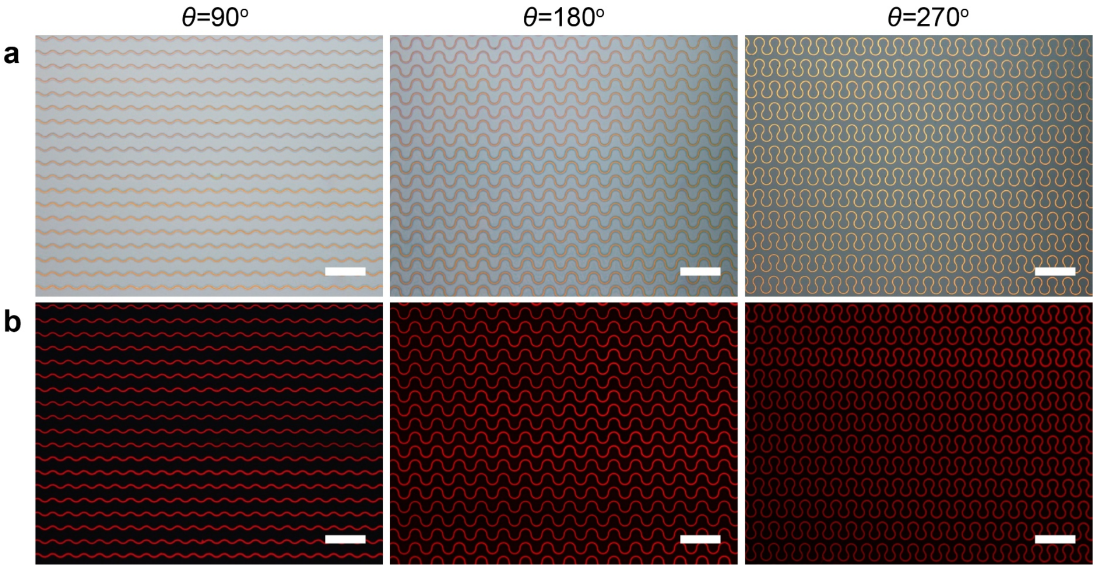


**Supplementary Fig. 17 | Large-area bright-field optical and fluorescent microscope images of curvilinear P3HT microstructure arrays. a**, Optical microscope image and **b**, fluorescent microscope images of curvilinear P3HT microstructure arrays with arc central angle of $\theta$ = 90°, 180° and 270°, and radius of $R$ = 15 μm. All scale bars, 100 μm.

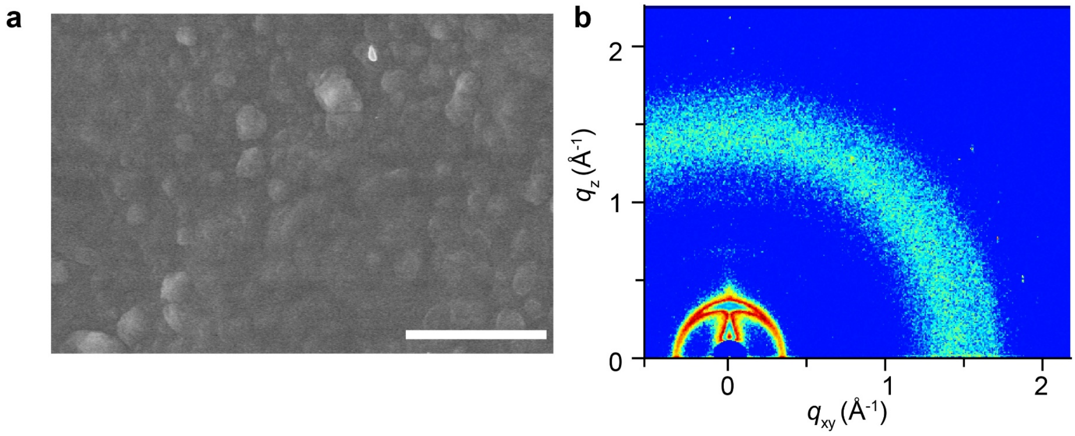


**Supplementary Fig. 18 | Morphological and crystallographic characterization of spin-coated P3HT film. a**, SEM image and **b**, GIWAXS data of spin-coated P3HT thin film. The toluene solution of P3HT polymer with the concentration of 5 mg mL$^{-1}$ was dropped onto the substrate and spin-coating at 1000 rpm for 60 s at room temperature to fabricate the polymer thin film. Scale bar: 10 μm.

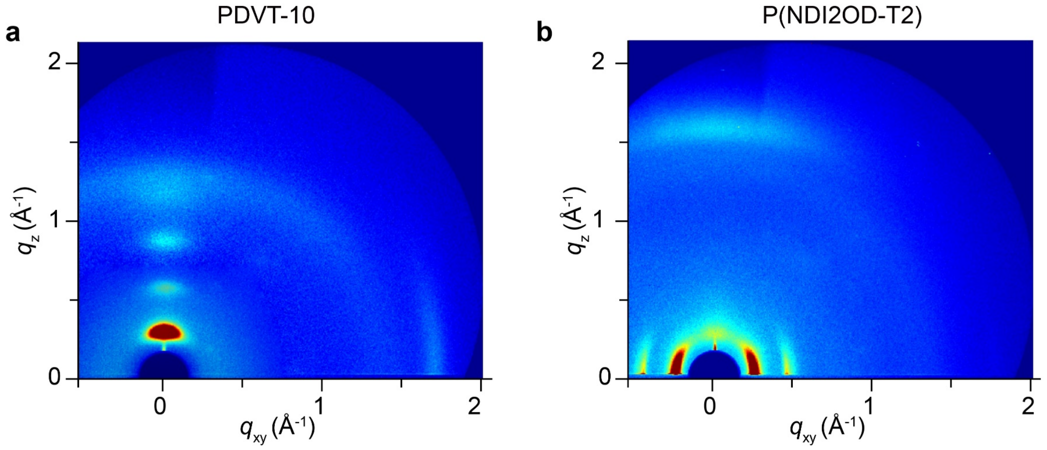


**Supplementary Fig. 19 | Crystallographic characterization of curvilinear conjugated polymer microstructure arrays.** GIWAXS data of **a** PDVT-10 and **b** P(NDI2OD-T2) organic semiconductor curvilinear microstructure arrays.

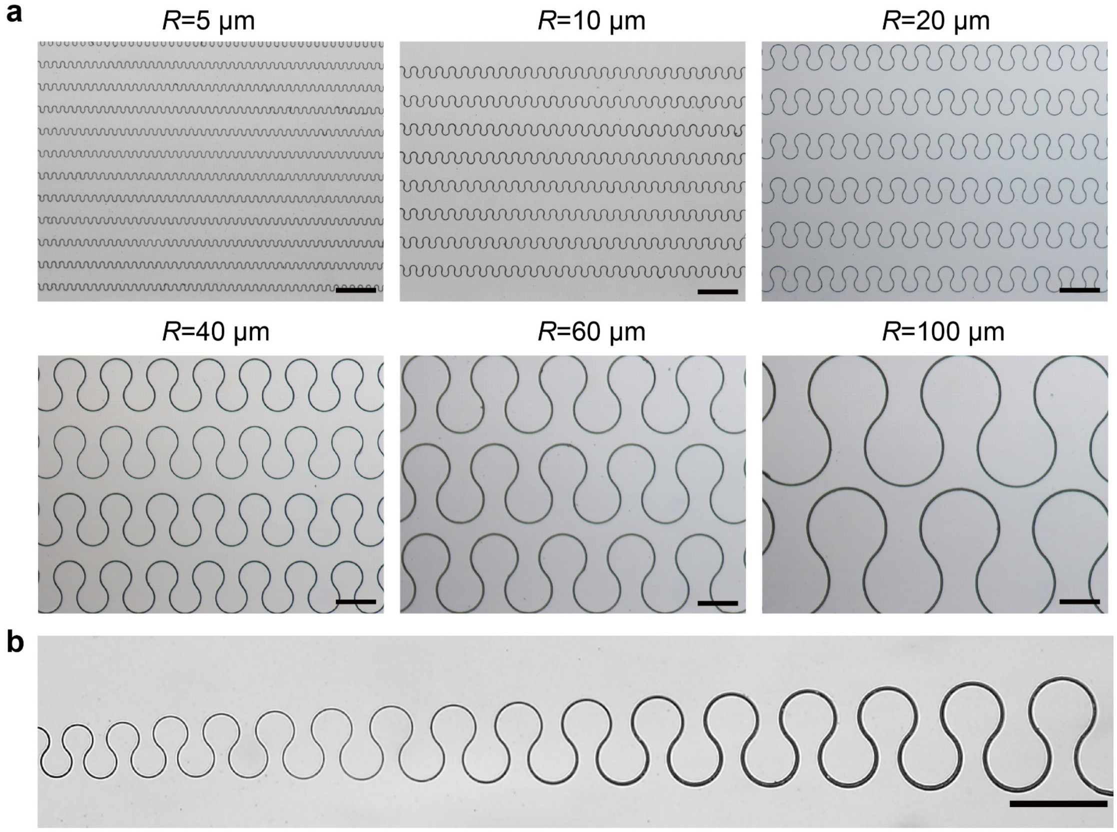


**Supplementary Fig. 20 | Optical micrographs of curvilinear microstructures with different geometrical parameters. a**, Representative microscope image of curvilinear microstructures with central arc angle of $\theta = 270°$, radius ranging from 5 to 100 μm. **b**, Representative microscope image of curvilinear microstructures with the incremental radius. All scale bars, 100 μm.

**Supplementary Note 2. Theoretical analysis for the morphology of as-fabricated curvilinear organic microstructures**

The width of curvilinear organic microstructure ($W_S$) depends on the mass of assembled organic polymer per unit area ($M_S \sim H_S \cdot L_S \cdot W_S$), where $H_S$ and $L_S$ are the height and length of curvilinear polymer microstructures, respectively. For the case of arc angle $\theta$ = 270°, $L_S = \frac{3}{4} \cdot 2\pi R$, where $R$ is the radius of arc. $H_S$ mainly depends on the applied pressure between micropillars and the substrate. We then obtain the following expression:

$$M_S \sim \frac{3}{2}\pi R \cdot W_S \tag{4}$$

The assembled mass of organic polymer proportion to the volume of polymer solution pinned in the inner arc ($V_P$) and the concentration of polymer solution ($C_P$). For the case of arc angle $\theta$ = 270°, $V_P = \frac{3}{4}\pi R^2 + \frac{R^2}{2}$. Then,

$$M_S \sim (\frac{3}{4}\pi R^2 + \frac{R^2}{2}) \cdot C_P \tag{5}$$

Together with formula (1) and (2), a simply equation for $W_S$ can be written as follows:

$$W_S \sim R \cdot C_P \tag{6}$$

From equation (3), we can understand that the parameter $R$ is a key factor to influence the width of curvilinear polymer microstructures.

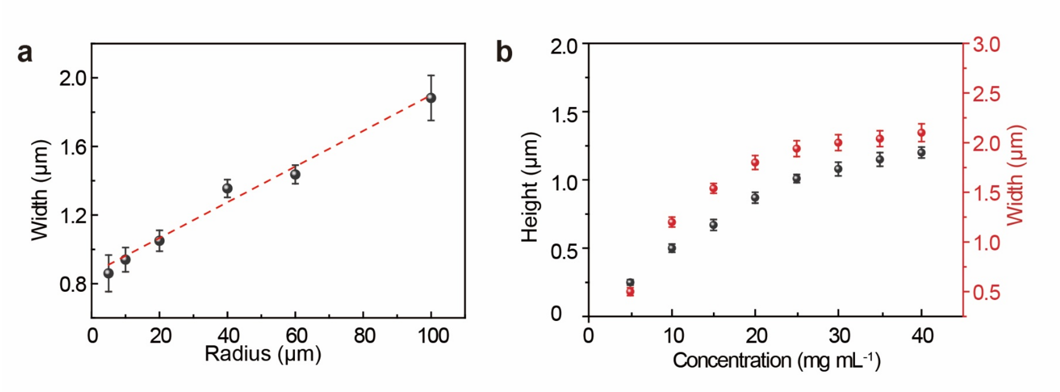


**Supplementary Fig. 21 | Statistical heights and widths of curvilinear microstructures.** Statistical morphological data of curvilinear P3HT microstructures with central arc angle of $\theta$ = 270° influenced by **a**, arc radius and **b**, solution concentrations.

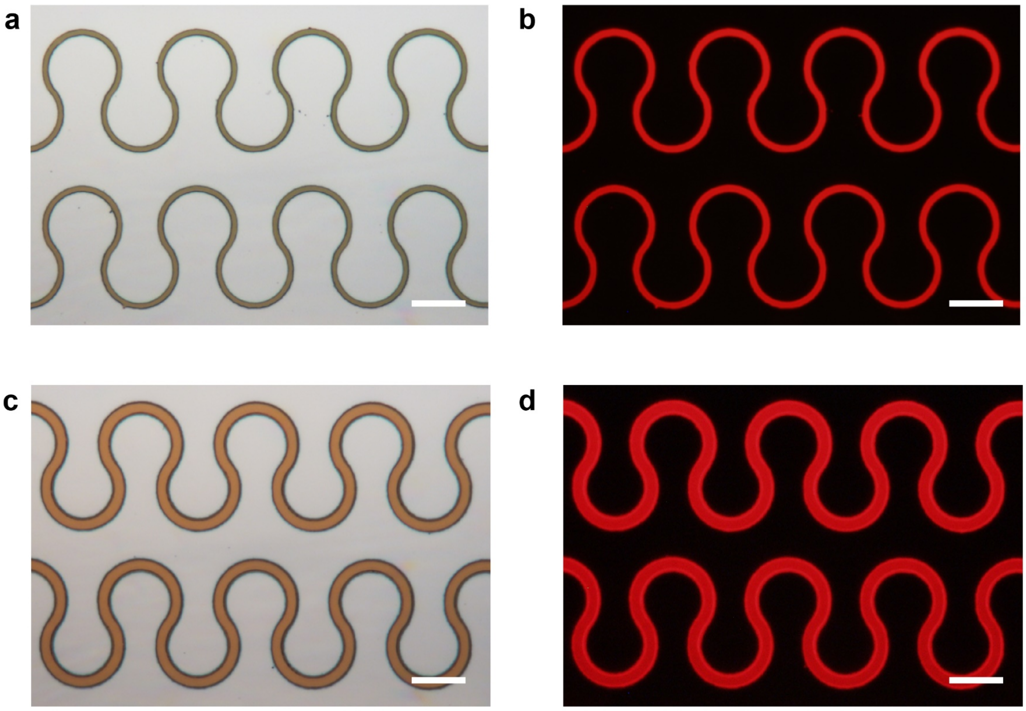


**Supplementary Fig. 22 | Curvilinear P3HT microstructure arrays with tunable width. a, c** Optical microscope images of curvilinear microstructure arrays fabricated by micropillar templates with the radius of $R$ = 10 μm, and width of 2 μm and 4 μm, respectively. **b, d** In-situ fluorescent microscope images of polymer arrays in **a** and **c**, respectively. All scale bars, 10 μm.

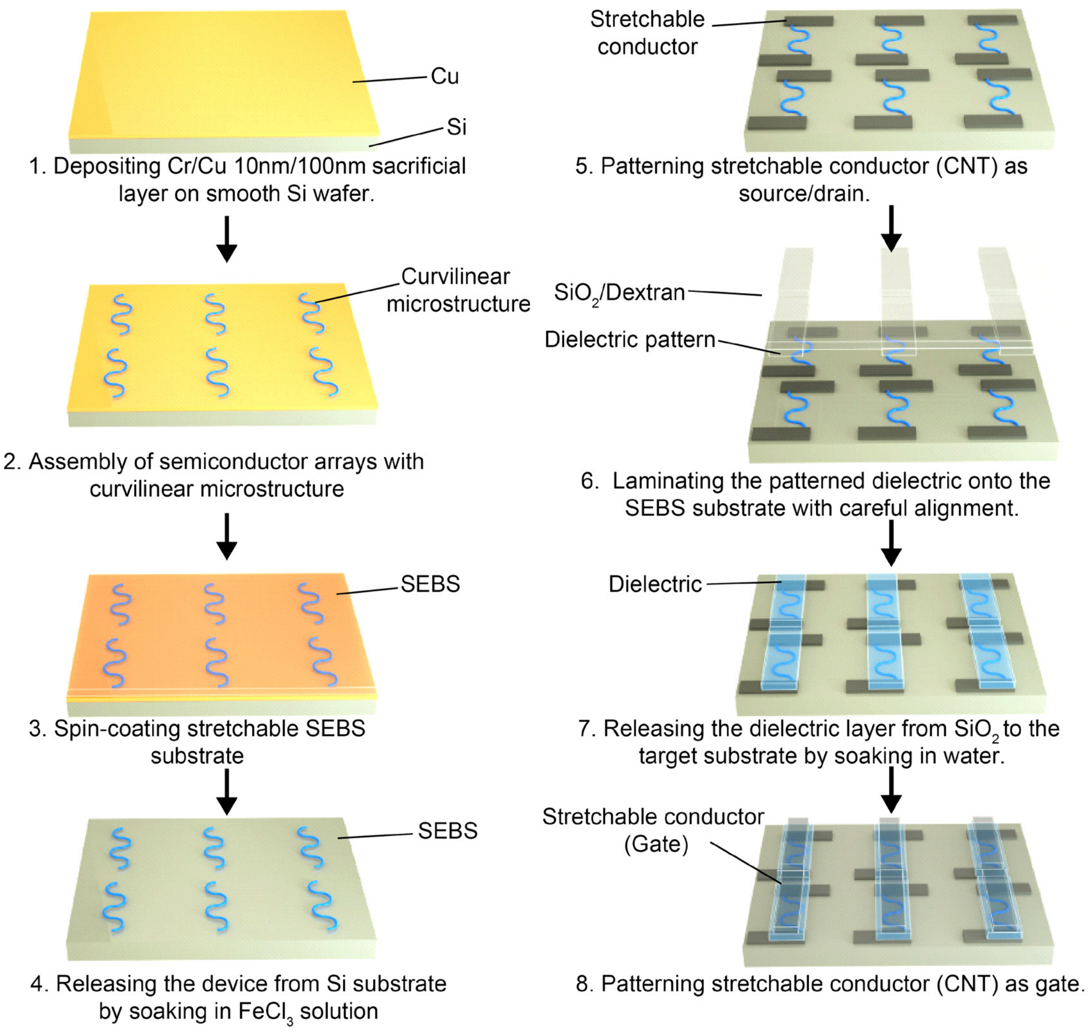


**Supplementary Fig. 23 | Schematic diagrams of fabricating intrinsically stretchable transistor arrays.** Si wafer coated with a sacrificial layer (100 nm Cu) was used as the target substrate to assemble the curvilinear organic microstructure arrays. Next, a stretchable SEBS layer was spin-coated on the substrate surface. The whole sample was then soaked in the $FeCl_3$ solution to peel curvilinear microstructure arrays from the rigid substrate. The stretchable conductor CNTs were selectively deposited as not only the patterned source and drain electrodes but also the wires for interconnecting adjacent devices. A stretchable SEBS dielectric layer was spin-coated onto a glass slide coated with water-soluble sacrificial dextran layer, then patterned by the laser irradiation. Finally, the patterned gate electrodes were selectively deposited to finish the fabrication of transistor devices.

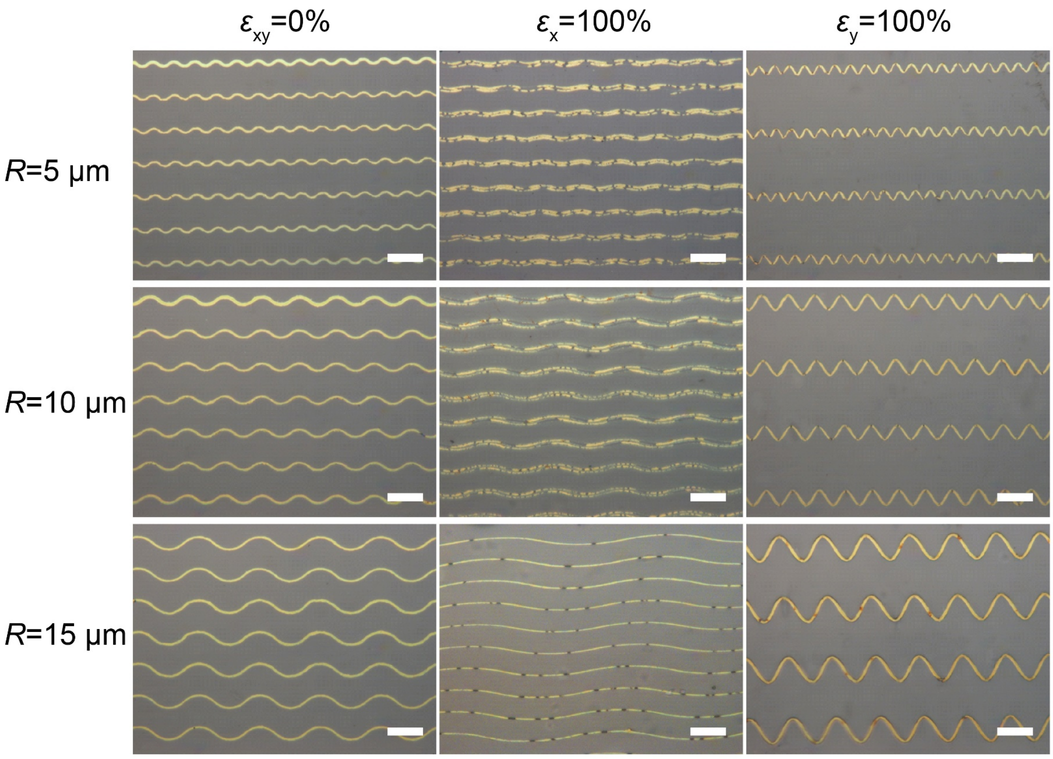


**Supplementary Fig. 24 | Mechanical tests of curvilinear P3HT microstructure arrays with central angle of $\theta$ = 90°.** Optical microscope images of the deformable curvilinear P3HT microstructure arrays ($\theta$ = 90° and $R$ = 5, 10 and 15 μm) on the SEBS substrate in the unstretched state ($\varepsilon_{xy}$ = 0%) and at 100% applied parallel ($\varepsilon_x$ = 100%) and perpendicular ($\varepsilon_y$ = 100%) strain. All scale bars, 20 μm.

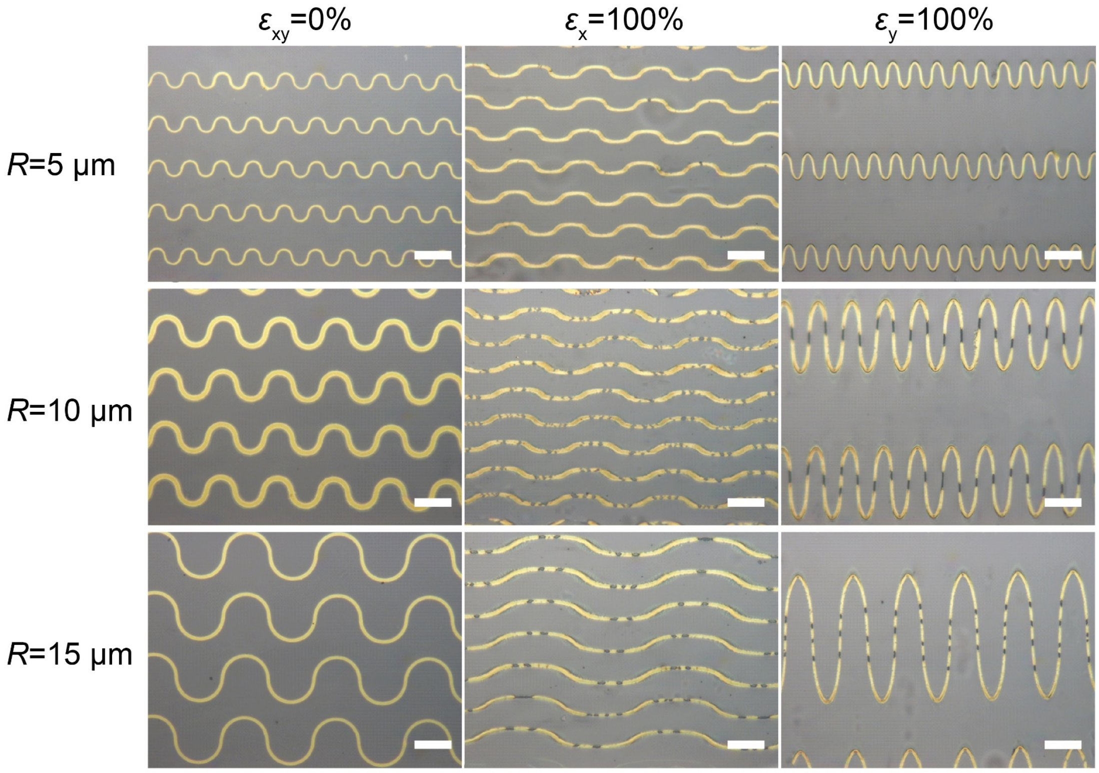


**Supplementary Fig. 25 | Mechanical tests of curvilinear P3HT microstructure arrays with central angle of $\theta$ = 180°.** Optical microscope images of the deformable curvilinear P3HT microstructure arrays ($\theta$ = 180° and $R$ = 5, 10 and 15 μm) on the SEBS substrate in the unstretched state ($\varepsilon_{xy}$ = 0%) and at 100% applied parallel ($\varepsilon_{x}$ = 100%) and perpendicular ($\varepsilon_{y}$ = 100%) strain. All scale bars, 20 μm.

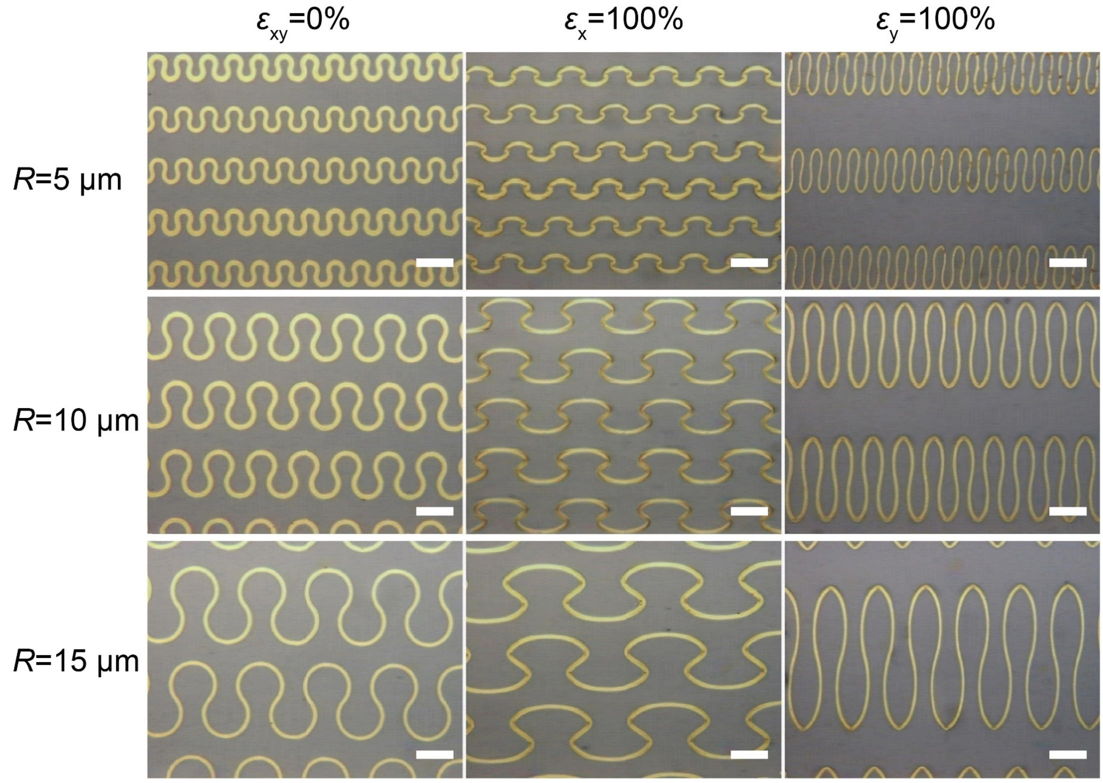


**Supplementary Fig. 26 | Mechanical tests of curvilinear P3HT microstructure arrays with central angle of *θ* = 270°.** Optical microscope images of the deformable curvilinear P3HT microstructure arrays ($\theta$ = 270° and $R$ = 5, 10 and 15 μm) on the SEBS substrate in the unstretched state ($\varepsilon_{xy}$ = 0%) and at 100% applied parallel ($\varepsilon_x$ = 100%) and perpendicular ($\varepsilon_y$ = 100%) strain. All scale bars, 20 μm.

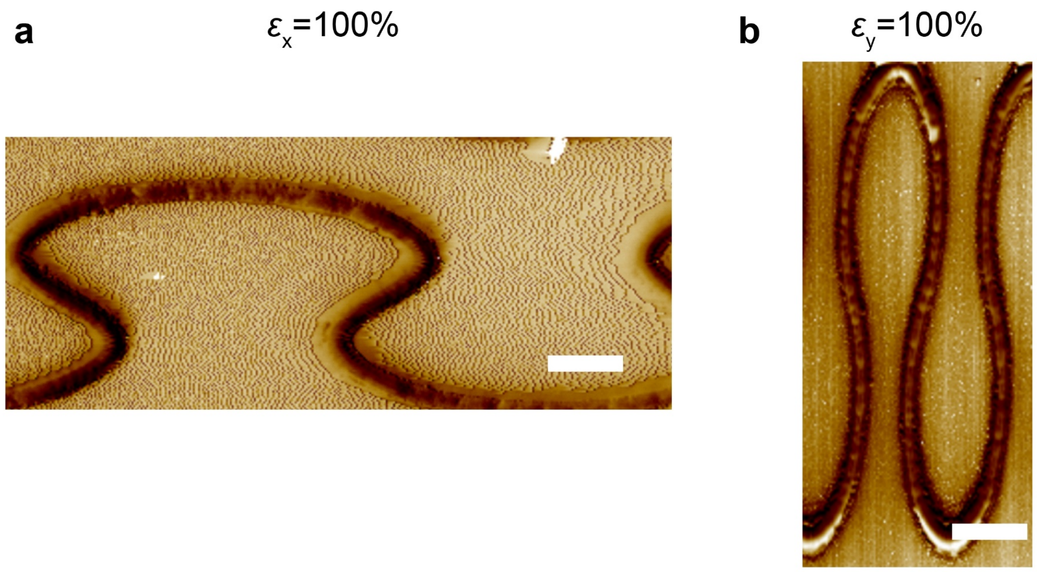


**Supplementary Fig. 27 | AFM phase images of P3HT curvilinear microstructures.** AFM phase images of polymer microstructures at **a**, 100% applied parallel ($\varepsilon_x = 100\%$) and **b**, perpendicular ($\varepsilon_y = 100\%$) strain, respectively. All scale bars, 20 µm.

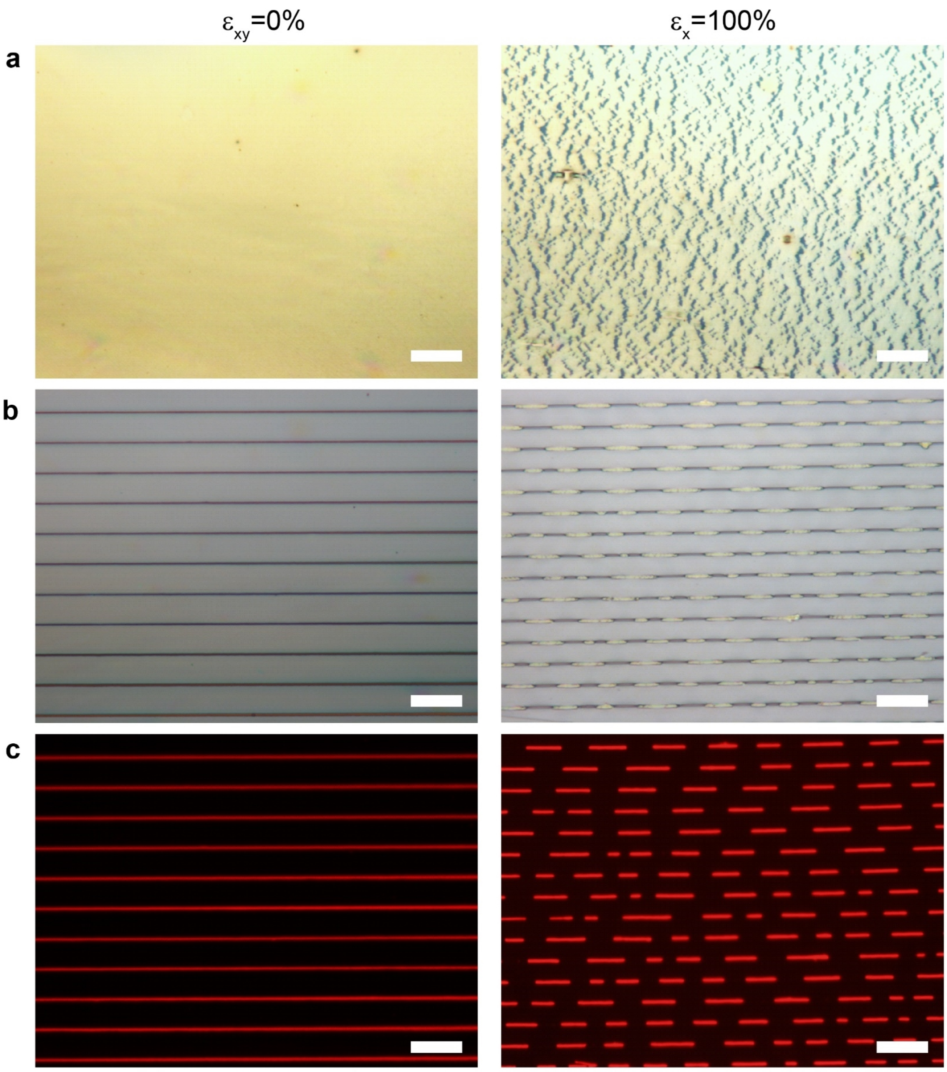


**Supplementary Fig. 28 | Mechanical tests of polymer spin-coating thin film and straight nanowire arrays. a**, Optical microscope images of spin-coating P3HT thin film in the unstretched state ($\varepsilon_{xy}$ = 0%) and at 100% applied parallel ($\varepsilon_x$ = 100%) strain. **b**, Optical microscope images and **c**, fluorescent microscope images of P3HT straight nanowire arrays on the SEBS substrate in the unstretched state ($\varepsilon_{xy}$ = 0%) and at 100% applied parallel ($\varepsilon_x$ = 100%) strain. All scale bars, 20 μm.

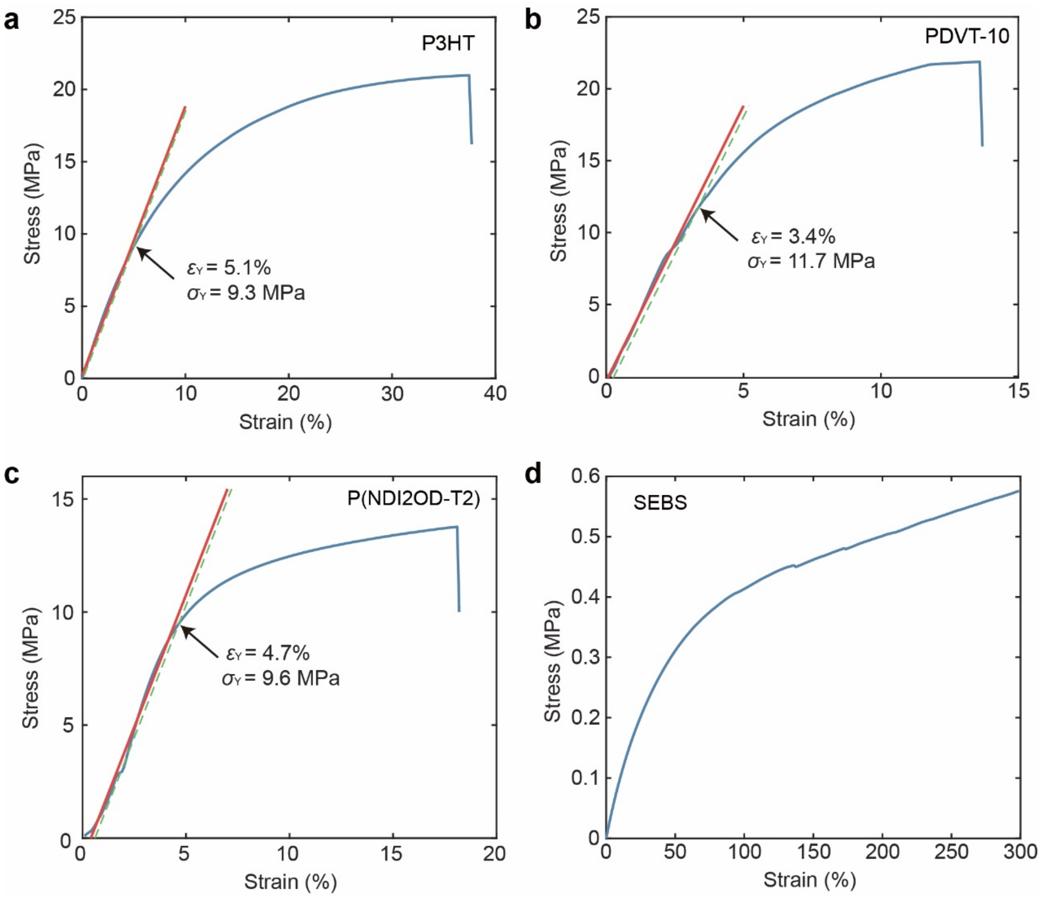


**Supplementary Fig. 29 | Tensile stress-strain curves of a, PDVT-10, b, P(NDI2OD-T2), c, P3HT, d, SEBS.** All stress-strain curves are measured on the bulk polymer belts. The yield strain $\varepsilon_Y$ and stress $\sigma_Y$ are defined as the strain and stress where the strain deviates 0.2% from the linearity.

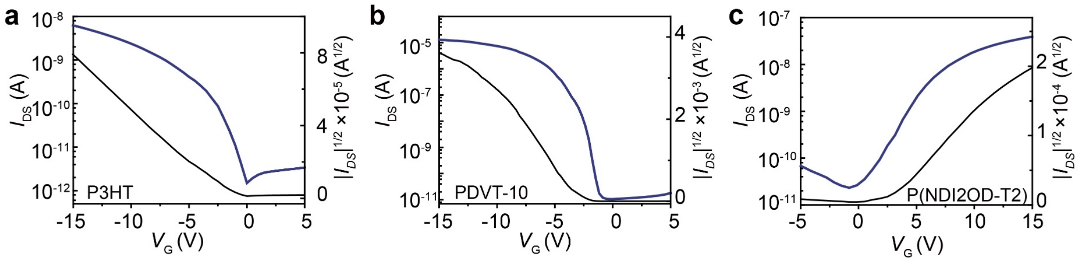


**Supplementary Fig. 30 | Electrical performance of OFET devices based on spin-coated thin film.** Transfer curves obtained from thin-film OFETs with three polymers.

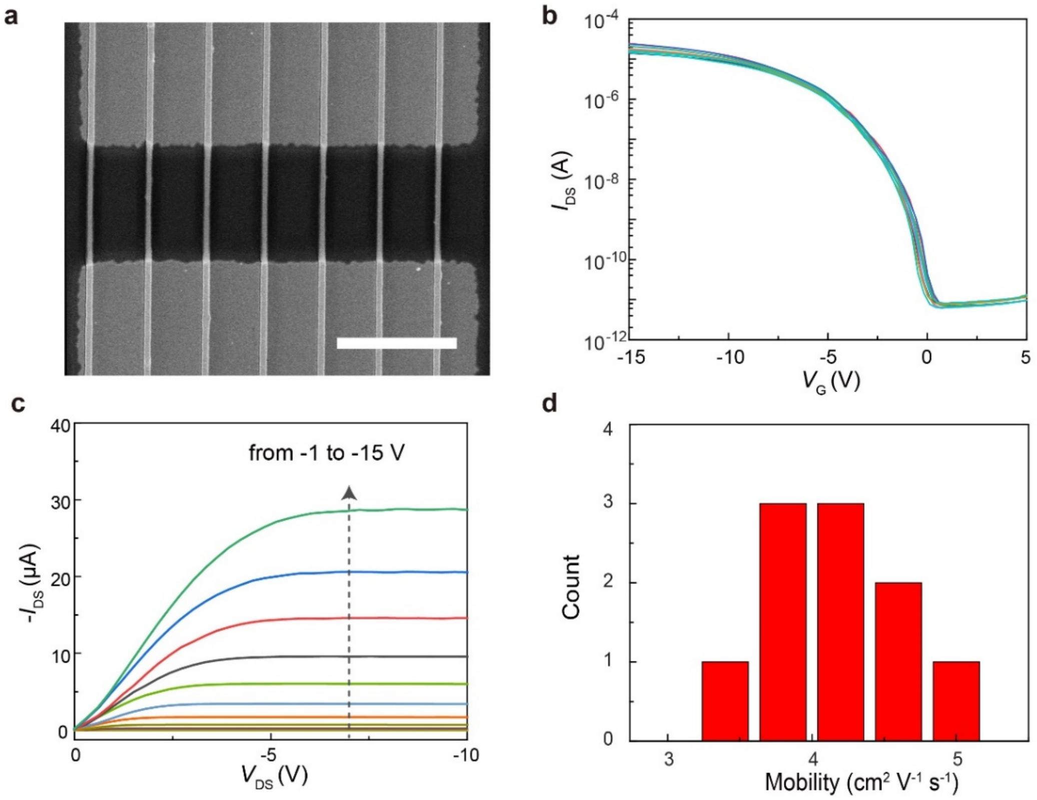


**Supplementary Fig. 31. | Field-effect mobility measurements based on straight polymer wire**. **a**, SEM image of a device based on straight wires of PDVT-10. Scale bar, 10 μm. The straight wires were fabricated by employing line-shape micropillar template followed by evaporating 5/50 nm Cr/Au as electrodes. **b**, Representative transfer curves from ten different devices constructed by straight PDVT-10 wires. A $V_{DS}$ of -10 V was applied for enabling the operation of devices in saturation region. **c**, Output curves of straight PDVT-10 wires. **d**, Statistics of mobility from ten devices.

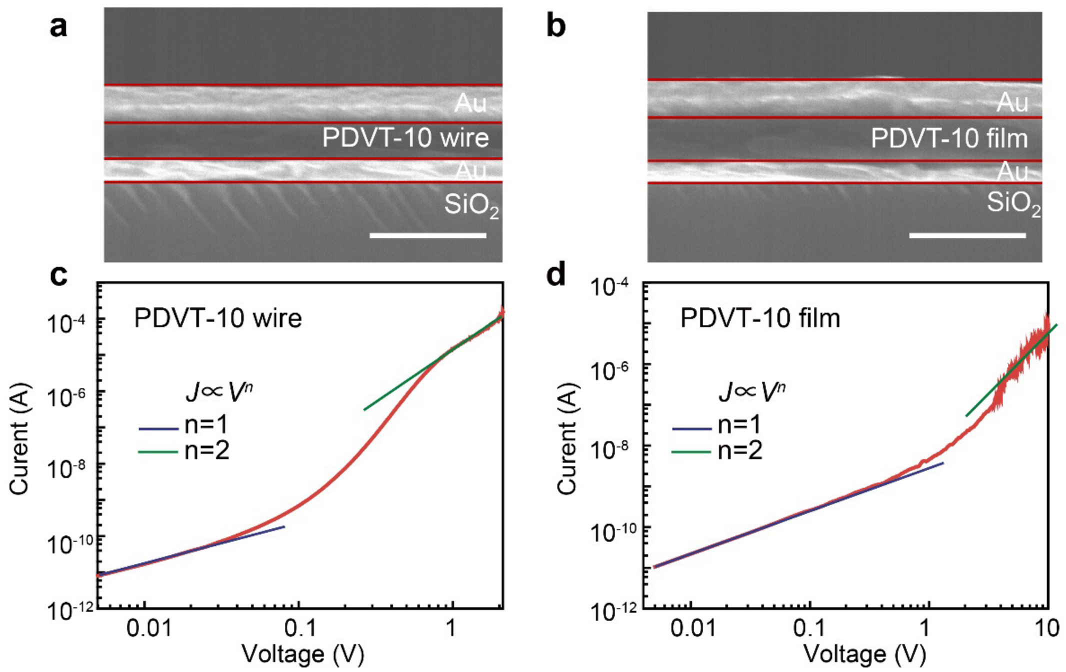


**Supplementary Fig. 32 | Space charge limited current measurements for extraction of carrier mobility**. SEM image of cross section of the hole-only devices constructed by (**a**) assembled microwire, and (**b**) spin-coated thin film. SCLC curves of (**c**) assembled microwire and (**d**) spin-coated thin film. Scale bars, 500 nm.

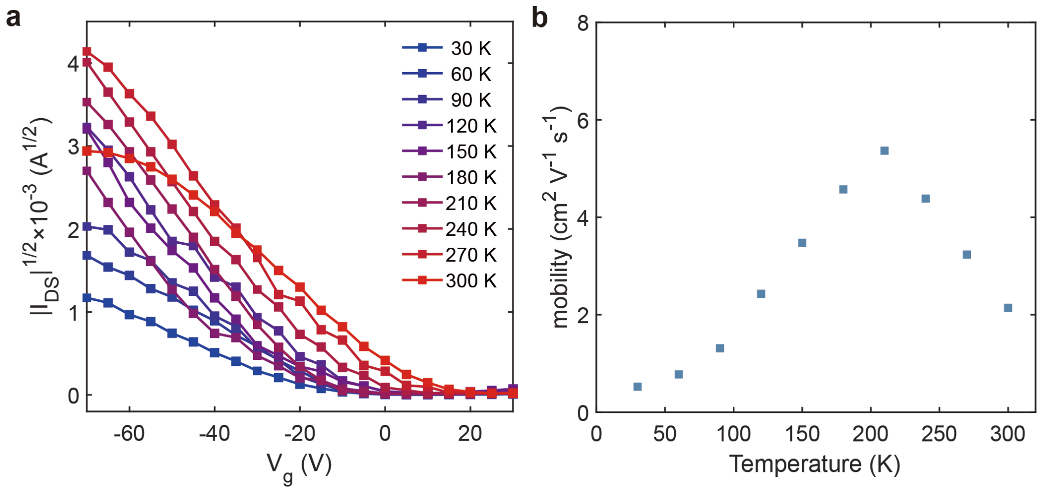


**Supplementary Fig. 33 | Temperature dependent field-effect mobility measurements on the assembled curvilinear microwires**. **a**, Transfer curves of curvilinear microwires at temperatures ranging from 30 to 300 K. To exclude the influence of traps at semiconductor-oxide interfaces, OTS-modified $SiO_2$ (300 nm in thickness) was employed as gate dielectric for the temperature-dependent measurements[1]. **b**, Mobility as a function of temperature. The negative temperature coefficient of mobility as the temperature above 210 K indicates the band-like transport.

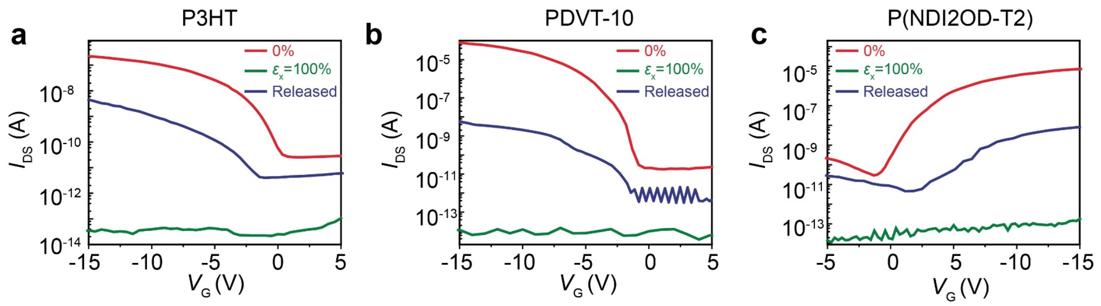


**Supplementary Fig. 34 | Electrical performance and stretchability of OFET devices based on straight polymer wires**. Transfer curves obtained from OFET based on line-shaped organic semiconductor at initial state, 100% strain parallel to the charge transport direction and after releasing back, respectively.

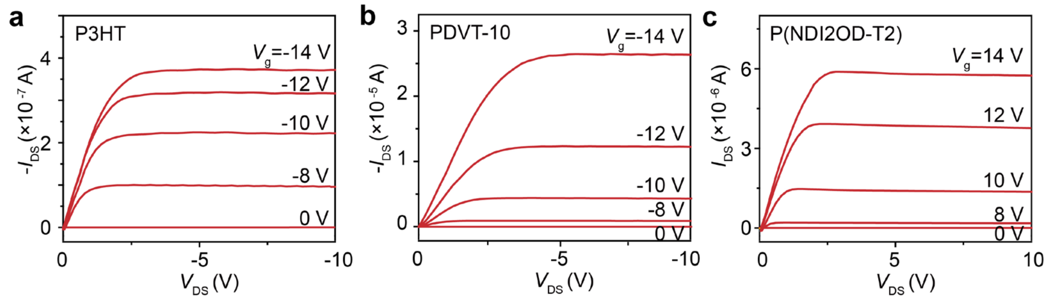


**Supplementary Fig. 35 | Output curves of OFETs without applied strain.** Output curves of OFETs with the active layers of **a**, P3HT, **b**, PDVT-10 and **c**, P(NDI2OD-T2).

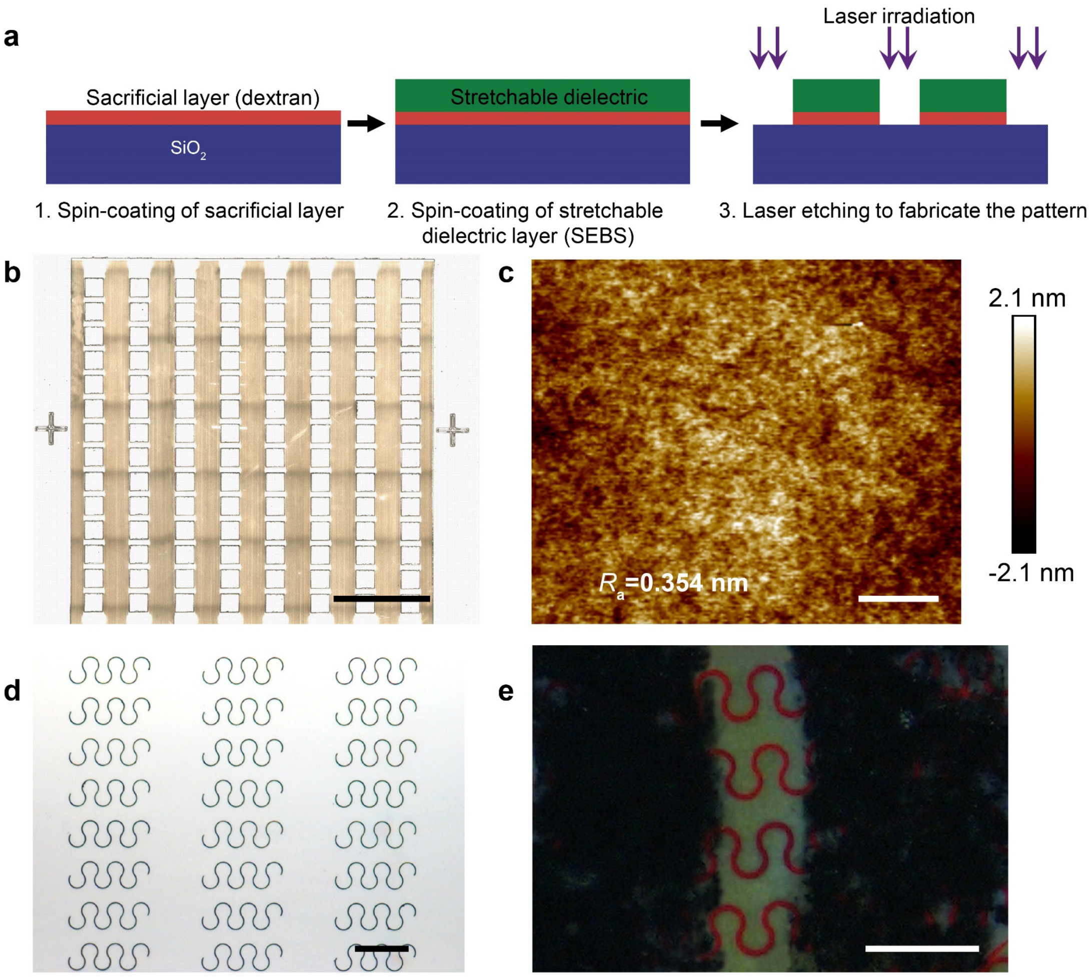


**Supplementary Fig. 36 | Fabrication of intrinsically stretchable transistor arrays.** **a**, Schematic diagrams of the fabrication process of patterning the SEBS dielectric layer. **b**, Optical microscope image of the patterned SEBS dielectric layer fabricated by the laser irradiation. **c**, AFM image of the SEBS dielectric layer, showing a low surface roughness of $R_a$=0.354 nm. **d**, Optical microscope image of discrete curvilinear polymer microstructure arrays. **e**, Fluorescent microscope image of the active channel (P3HT) and the source/drain electrodes (CNT). Scale bars: **b**, 2 mm, **c**, 20 µm, **d**, 50 µm, **e**, 50 µm.

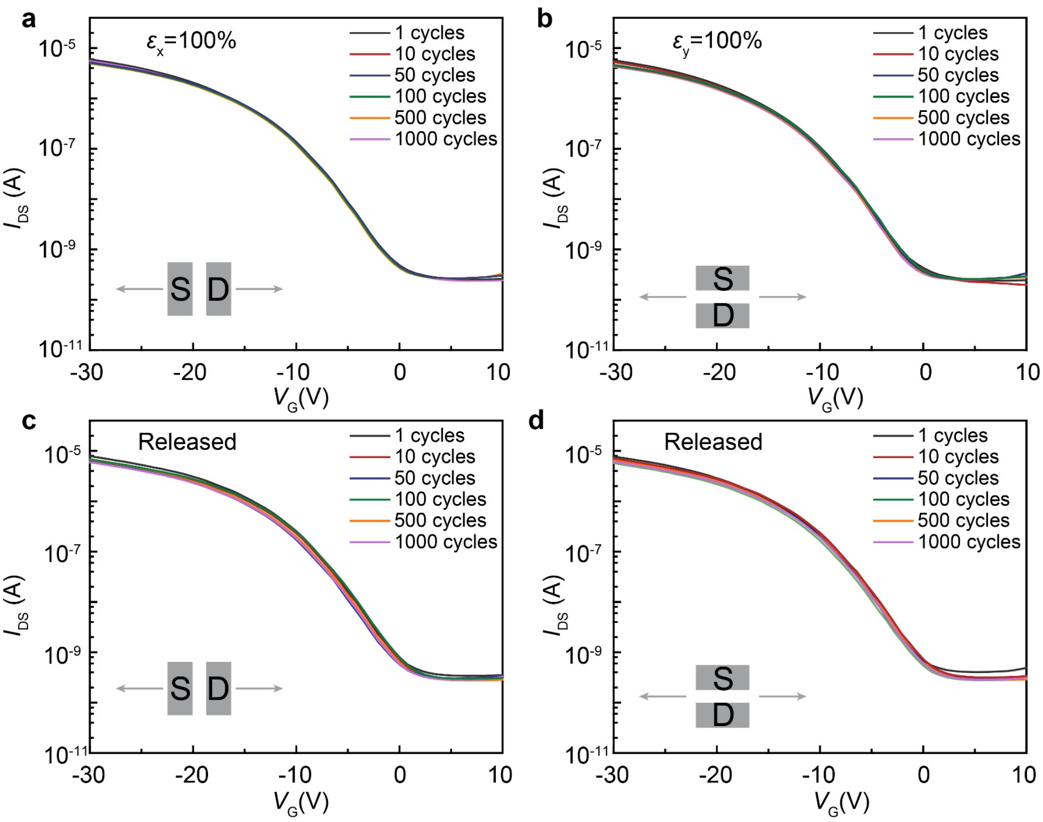


**Supplementary Fig. 37 | Transfer curves from the fully stretchable OFET array under the repeated stretching to 50% strain for 1000 cycles**. **a**, **b**, Transfer curves obtained from OFET under 50% strain along both (**a**) horizontal and (**b**) vertical directions. **c**, **d**, Transfer curves obtained from OFET released back from stretching along both (**c**) horizontal and (**d**) vertical directions.

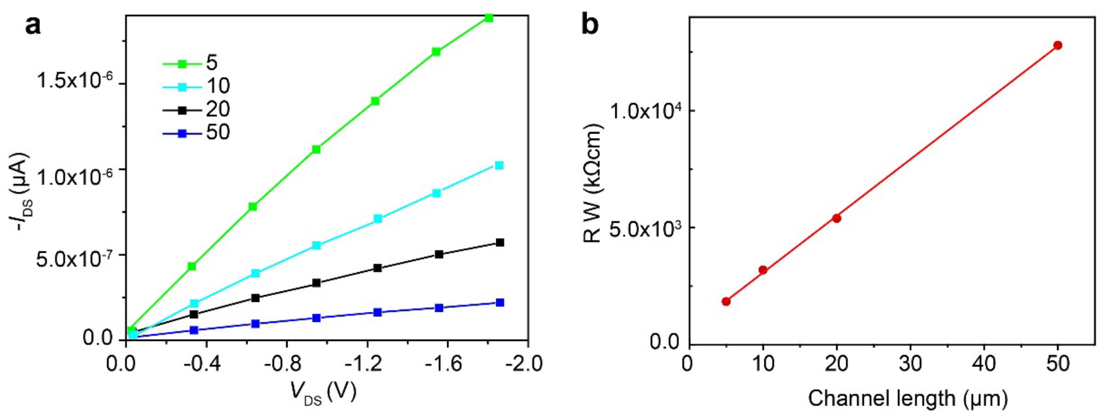


**Supplementary Fig. 38 | Characterizations of the contact resistance in the OFFTs.** **a**, I-V curves of pairs of electrodes with channel lengths ranging from 5 to 50 μm from the transistors. Straight microwires were employed to enable the accurate measurements of channel length. The drain voltage ranges from 0 to -2 V and the gate voltage is fixed at -15V. **b**, Width-normalized total device resistance $R_{tot}W$ as a function of channel length. The width-normalized contact resistance of 0.61 kΩ·cm is extracted from the intercept at the $R_{tot}W$ axis.

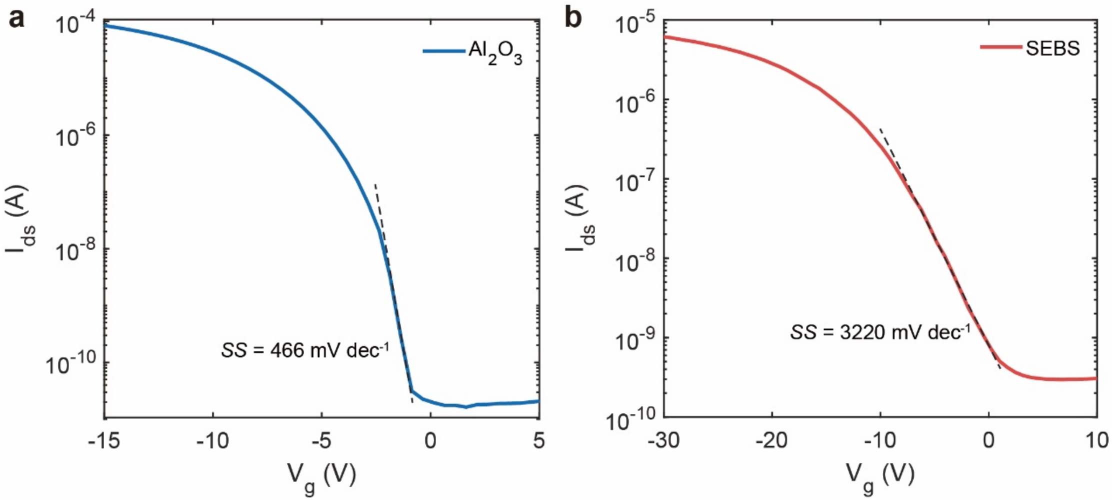


**Supplementary Fig. 39 | Subthreshold slope of FETs** based on **a**, high-k $Al_2O_3$, and **b**, elastic SEBS gate dielectrics. The *SS* is correlated with the interfacial traps by $SS = mk_BT/e$, where $k_B$ is the Boltzmann constant, $T$ is the temperature, $e$ is the elementary charge, and $m$ is the body factor, which can be expressed as, $m = \left(1 + \frac{C_S}{C_{DE}} + \frac{C_{IT}}{C_{DE}}\right)$, where $C_S$, $C_{DE}$ and $C_{IT}$ are the capacitance originating from semiconductor, dielectrics and interfacial traps, respectively. *SS* of 446 and 3220 mV $dec^{-1}$ can be observed, indicating that $(C_S + C_{IT})$ equals to $6.8C_{Al2O3}$ and $52.7C_{SEBS}$, for $Al_2O_3$ and SEBS dielectrics, respectively. The capacitance of $Al_2O_3$ $C_{Al2O3}$ is $1.33\times10^{-7}$ F $cm^{-2}$ by considering its dielectric constant of 7.5 and thickness of 50 nm, while the capacitance of SEBS $C_{SEBS}$ in 1.2 μm thickness is $1.5\times10^{-9}$ F $cm^{-2}$. Thus, the $(C_S + C_{IT})$ values are determined as $9.0\times10^{-7}$ and $7.9\times10^{-8}$ F $cm^{-2}$ for $Al_2O_3$ and SEBS dielectrics, respectively.

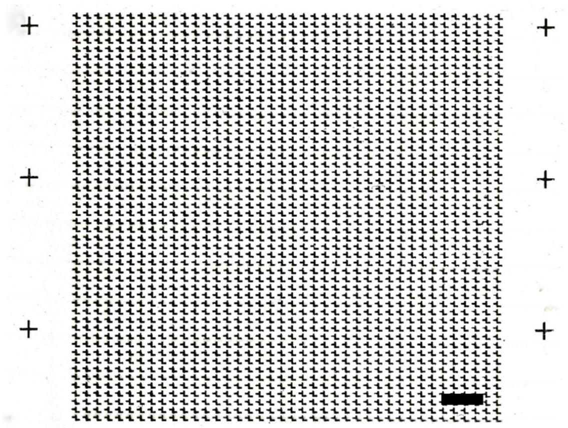

**Supplementary Fig. 40 | Optical microscope image of 2000 stretchable transistor arrays.** Scale bar, 2 mm.

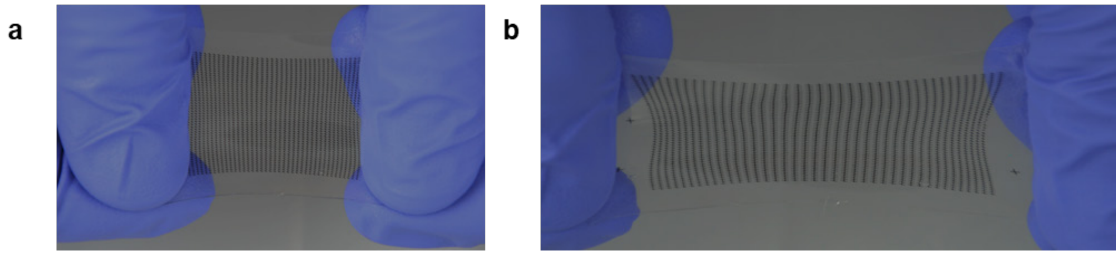


**Supplementary Fig. 41 | Digital photographs of stretchable transistor arrays** at **a**, 0% and **b**, 100% strain.

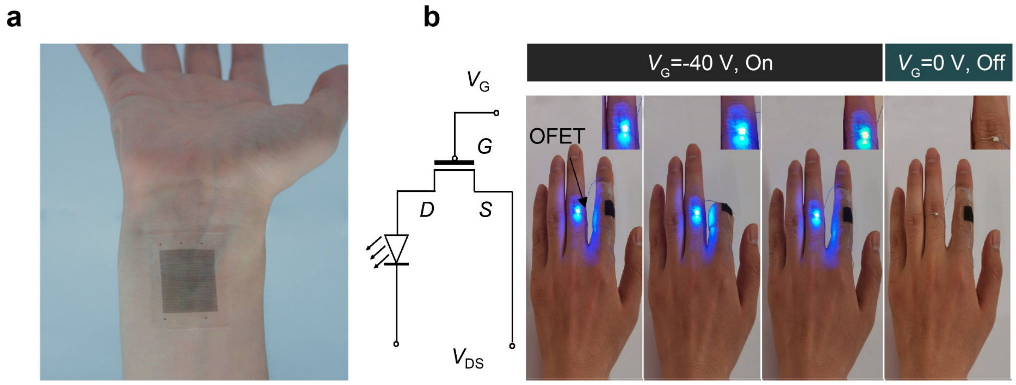


**Supplementary Fig. 42 | Stretchable transistor arrays for functional skin electronics. a**, A large-scale integrated device with 2000 stretchable transistors in an area of around 3 × 3 cm$^2$, which is attached on a human inner wrist. **b**, A finger-wearable mechanical sensor constructed by fully stretchable transistor arrays as the controller and an LED as the indicator, demonstrating the function for indicating the finger bending.

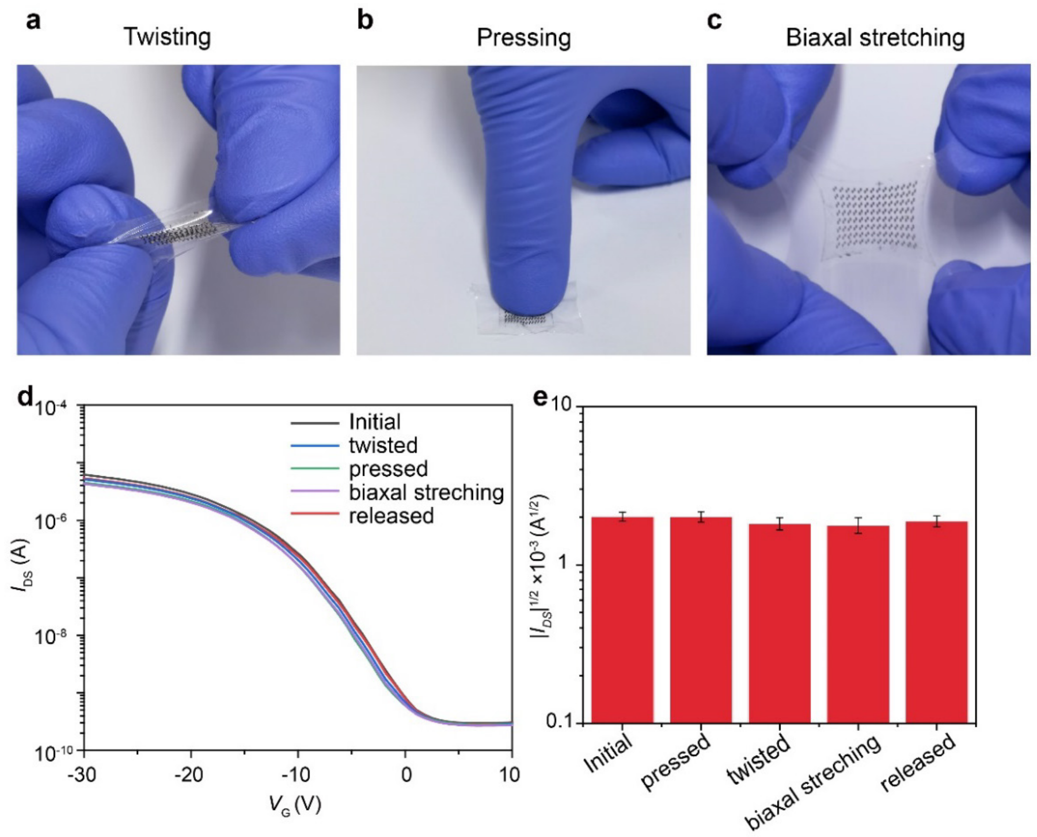


**Supplementary Fig. 43 | Electronic performances of the OFETs under various deformation situations.** a-c, Pictures showing the stretchable transistor arrays under various deformations, including twisting, pressing and biaxial stretching. d, Transfer curves in the primary state, and after twisting, pressing, biaxial stretching and releasing. e, Mobilities obtained after various deformation.

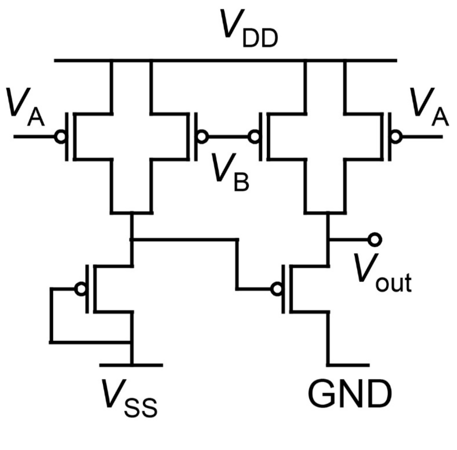


**Supplementary Fig. 44 | Circuit diagrams of the intrinsically stretchable NAND gate.**

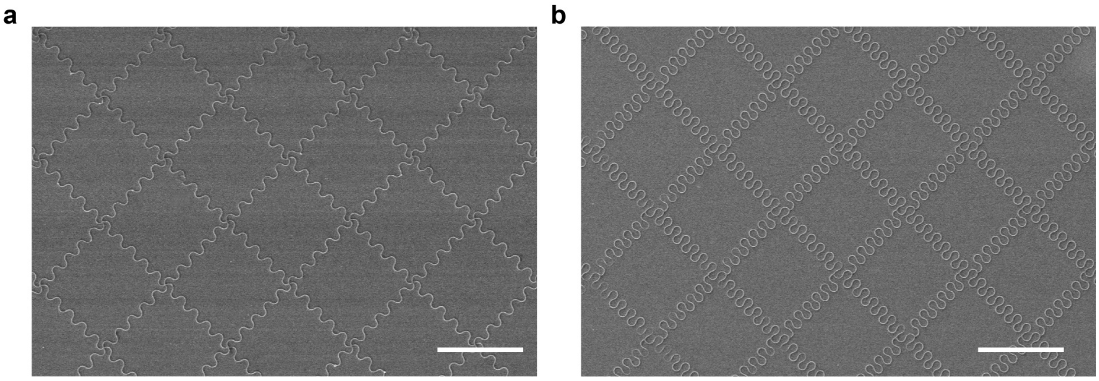


**Supplementary Fig. 45 | SEM images of kirigmi-inspired 2D polymer curvilinear networks.** Organic 2D curvilinear networks constructed by curvilinear polymer microstructures with the central angle of **a**, 180°, **b**, 270°. All scale bars: 50 μm.

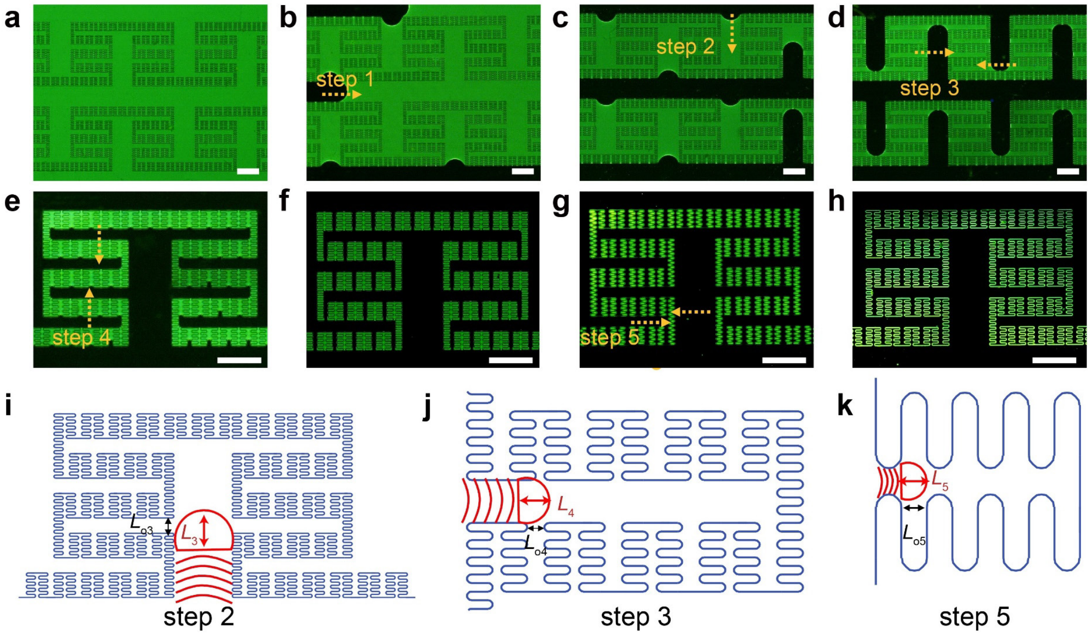


**Supplementary Fig. 46 | Dewetting processes in multi-order hierarchical fractal structured system. a**–**h**, Fluorescent microscope observation of the dewetting process in multi-order hierarchical fractal structured system. **i**–**k**, Schematic illustration of the gas flow behavior at the curvilinear opening in the dewetting process of step 2-4. In good accordance with above mentioned equation, if the length of fluid front $L$ is larger than curvilinear opening $L_o$, the liquid is pinned into the groove. All scale bars, 200 μm.

**Supplementary table 1 | Performance comparison of our stretchable transistors with previously reported fully stretchable transistors.**

| Material | On/off ratio | Mobility at zero strain ($cm^2\ V^{-1}\ s^{-1}$) | Endurable strain | Mobility at endurable strain | Device scale | Ref |
|---|---|---|---|---|---|---|
| PDVT-10 | $10^4$ | 2.4 ± 0.3 | 100% | 2.2 ± 0.1 | 4-inch wafer scale | This work |
| DPP2TTVT-PDCA | $10^4$ | 0.821 ± 0.105 | 100% | 0.99 | 6,300 devices | 2 |
| P3HT | 591 ± 461 | 0.034 ± 0.016 | 100% | 0.005 | Single device | 3 |
| FT4-DPP: PEO | $10^3$ | 0.78 ± 0.21 | 100% | 0.3 ± 0.07 | Single device | 4 |
| DPP | $10^6$ | 1.3 | 100% | 1.1 | 25 devices | 5 |
| P3HT | $10^4$ | 0.006 | 50% | 0.002 | Single device | 6 |
| DPPT-TT | $10^4$ | 0.59 | 100% | 0.55 | Single device | 7 |
| DPPDTSE | $10^4$ | 1.5 | 100% | 1.5 | 20 devices | 8 |
| C12-DPP | $10^4$ | 0.46 | 100% | 0.36 | Single device | 9 |
| TTA-DPP | $1.3\times10^3$ | 0.22 | 100% | 0.07 | Single device | 10 |
| PII2T | $10^3$ | 0.6 | 100% | 0.1 | 25 devices | 11 |

## Supplementary References


1. Sirringhaus, H. Reliability of organic field-effect transistors. *Adv. Mater.* **21**, 3859-3873, (2009).
2. Wang, S. et al. Skin electronics from scalable fabrication of an intrinsically stretchable transistor array. *Nature* **555**, 83-88 (2018).
3. Chortos, A. et al. Highly stretchable transistors using a microcracked organic semiconductor. *Adv. mater.* **26**, 4253-4259 (2014).
4. Lee, Y. et al. Deformable organic nanowire field-effect transistors. *Adv. mater.* **30**, 1704401 (2018).
5. Oh, J.Y. et al. Intrinsically stretchable and healable semiconducting polymer for organic transistors. *Nature* **539**, 411-415 (2016).
6. Shin, M. et al. Polythiophene nanofibril bundles surface-embedded in elastomer: a route to a highly stretchable active channel layer. *Adv. Mater.* **27**, 1255-1261 (2015).
7. Xu, J. et al. Highly stretchable polymer semiconductor films through the nanoconfinement effect. *Science* **355**, 59-64 (2017).
8. Xu, J. et al. Multi-scale ordering in highly stretchable polymer semiconducting films. *Nat. Mater.* **18**, 594-601 (2019).
9. Mun, J. et al. Effect of nonconjugated spacers on mechanical properties of semiconducting polymers for stretchable transistors. *Adv. Funct. Mater.* **28**, 1804222 (2018).
10. Rao, Y.L. et al. Stretchable self-healing polymeric dielectrics cross-linked through metal-ligand coordination. *J. Am. Chem. Soc.* **138**, 6020-6027 (2016).
11. Liu, J. *et al.* Fully stretchable active-matrix organic light-emitting electrochemical cell array. *Nat. Commun.* **11**, 3362 (2020).
