# Supplementary material for: Wafer-scale integration of stretchable semiconducting polymer microstructures via capillary gradient
Source: Nat Commun. 2021 Dec 2;12:7038. doi: 10.1038/s41467-021-27370-w (PMC8640044; doi:10.1038/s41467-021-27370-w)
Supplement: Supplementary file 2 — Description of Additional Supplementary Files [file 41467_2021_27370_MOESM2_ESM.pdf]

Description of Additional Supplementary Files

File name: Supplementary Movie 1

Description: Fluid dynamics simulations of the dewetting process
